# Predicting progression to Alzheimer’s disease with human hippocampal progenitors exposed to serum

**DOI:** 10.1093/brain/awac472

**Published:** 2023-01-27

**Authors:** Aleksandra Maruszak, Edina Silajdžić, Hyunah Lee, Tytus Murphy, Benjamine Liu, Liu Shi, Chiara de Lucia, Abdel Douiri, Evgenia Salta, Alejo J Nevado, Charlotte E Teunissen, Pieter J Visser, Jack Price, Henrik Zetterberg, Simon Lovestone, Sandrine Thuret

**Affiliations:** Department of Basic and Clinical Neuroscience, Institute of Psychiatry Psychology & Neuroscience, King’s College London, London, SE5 9RX, UK; Department of Basic and Clinical Neuroscience, Institute of Psychiatry Psychology & Neuroscience, King’s College London, London, SE5 9RX, UK; Department of Basic and Clinical Neuroscience, Institute of Psychiatry Psychology & Neuroscience, King’s College London, London, SE5 9RX, UK; Department of Basic and Clinical Neuroscience, Institute of Psychiatry Psychology & Neuroscience, King’s College London, London, SE5 9RX, UK; Department of Psychiatry, University of Oxford, Oxford, OX3 7JX, UK; Department of Psychiatry, University of Oxford, Oxford, OX3 7JX, UK; Department of Basic and Clinical Neuroscience, Institute of Psychiatry Psychology & Neuroscience, King’s College London, London, SE5 9RX, UK; Department of Population Health Sciences, King's College London, London, SE1 1UL, UK; Netherlands Institute for Neuroscience, 1105 BA Amsterdam, The Netherlands; Neurochemistry Lab and Biobank, Department of Clinical Chemistry, Amsterdam Neuroscience, VU University Medical Center, 1007 MB Amsterdam, The Netherlands; Department of Psychiatry, University of Oxford, Oxford, OX3 7JX, UK; Neurochemistry Lab and Biobank, Department of Clinical Chemistry, Amsterdam Neuroscience, VU University Medical Center, 1007 MB Amsterdam, The Netherlands; Department of Psychiatry and Neuropsychology, Alzheimer Center Limburg, School for Mental Health and Neuroscience, Maastricht University, 6200 MD Maastricht, The Netherlands; Department of Neurology, Alzheimer Center, VU University Medical Center, 1081 HZ Amsterdam, The Netherlands; Department of Basic and Clinical Neuroscience, Institute of Psychiatry Psychology & Neuroscience, King’s College London, London, SE5 9RX, UK; Clinical Neurochemistry Laboratory, Sahlgrenska University Hospital, S-431 80 Mölndal, Sweden; Department of Neurodegenerative Disease, UCL Institute of Neurology, London, WC1N 3BG, UK; Institute of Neuroscience and Physiology, Department of Psychiatry and Neurochemistry, the Sahlgrenska Academy at the University of Gothenburg, S-431 80 Mölndal, Sweden; UK Dementia Research Institute at UCL, London, WC1E 6BT, UK; Department of Psychiatry, University of Oxford, Oxford, OX3 7JX, UK; Janssen Medical UK, B-2340 Beerse, Belgium; Department of Basic and Clinical Neuroscience, Institute of Psychiatry Psychology & Neuroscience, King’s College London, London, SE5 9RX, UK

**Keywords:** Alzheimer’s disease, prognostic biomarker, neurogenesis, hippocampal progenitors

## Abstract

Adult hippocampal neurogenesis is important for learning and memory and is altered early in Alzheimer’s disease. As hippocampal neurogenesis is modulated by the circulatory systemic environment, evaluating a proxy of how hippocampal neurogenesis is affected by the systemic milieu could serve as an early biomarker for Alzheimer’s disease progression. Here, we used an *in vitro* assay to model the impact of systemic environment on hippocampal neurogenesis. A human hippocampal progenitor cell line was treated with longitudinal serum samples from individuals with mild cognitive impairment, who either progressed to Alzheimer’s disease or remained cognitively stable. Mild cognitive impairment to Alzheimer’s disease progression was characterized most prominently with decreased proliferation, increased cell death and increased neurogenesis. A subset of ‘baseline’ cellular readouts together with education level were able to predict Alzheimer’s disease progression. The assay could provide a powerful platform for early prognosis, monitoring disease progression and further mechanistic studies.

## Teaser

Adult hippocampal neurogenesis is altered early in the course of Alzheimer’s disease. Maruszak *et al.* show that serum from patients with MCI who do versus do not progress to Alzheimer’s disease differentially affects the fate of hippocampal stem cells *in vitro*, suggesting that this assay could help predict disease progression.

## Introduction

Alzheimer’s disease is a progressive neurodegenerative condition without effective treatment options. Individuals diagnosed with mild cognitive impairment (MCI) are known to progress to Alzheimer’s disease at a significantly higher rate (10–15% in clinical studies, 5–10% in population studies) compared to cognitively healthy elderly people (1–2%).^1^ However, not all individuals with MCI develop Alzheimer’s disease, which calls for the need to develop an accurate estimation of how likely an individual with MCI is to progress to Alzheimer’s disease. Given the current consensus that putative Alzheimer’s disease-modifying therapies work best when administered during the preclinical stage, the estimation should be done preferably at the earliest stages of disease progression to maximize the success of intervention. Recently, several studies have suggested blood-based biomarkers as promising targets to monitor early disease progression and predict cognitive decline, and most of them are associated with well-established Alzheimer’s disease hallmarks.^2,3^ However, they provide limited information on how the systemic environment impact the brain at the cellular level, and this calls for a need to develop a biomarker that allows us to gain a better understanding of what occurs at the ‘cellular phase’ of early Alzheimer’s disease.^4^

Hippocampal neurogenesis (HN) occurs throughout life in the subgranular zone of the mammalian dentate gyrus. The hippocampal neurogenic niche is composed of hippocampal progenitor cells (HPCs), their progeny (i.e. neurons and glia), endothelial cells and a highly vascularized extracellular matrix.^5^ While the existence of neurogenesis in adult humans has been questioned,^6,7^ an overwhelming majority of the existing literature^8^ shows unequivocally that HN is a lifelong process that occurs in many mammalian species, including human and is important for hippocampus-dependent learning and memory.^9,10^

Interestingly, HN is highly sensitive to the circulatory systemic environment which is well-demonstrated by parabiosis experiments where the circulatory systems of two animals are surgically conjoined.^11,12^ Blood from young mice can exert a rejuvenating effect on the old animals’ cognition by improving HN,^13,14^ and vice versa.^11,15^ Moreover, interventions that target the systemic environment (i.e. drugs, exercise, diet) have been shown to modulate HN.^16–18^ Importantly, interventions like exercise^19,20^ and diet that ‘increase HN’ have been associated with ‘decreased Alzheimer’s disease risk’.^21,22^

Several post-mortem studies on human Alzheimer’s disease brains have recently demonstrated that significant changes in HN can be observed from as early as Braak stage II of Alzheimer’s disease,^23,24^ which is in line with rodent model studies where altered HN was indeed an early indication of Alzheimer’s disease progression.^25,26^ It is also worth noting that the hippocampus is one of the brain regions affected early on in Alzheimer’s disease, and its atrophy is significantly associated with memory loss and learning impairment.^27,28^ While this evidence collectively suggest that changes in HN can serve as a potential biomarker for early disease progression,^29–31^ neither rodent nor human studies have been in full agreement with regards to the directionality and magnitude of these changes. While most studies report a reduction of HN,^23,24,26^ some report an increase.^32–34^ Such discrepancy amongst existing studies suggests a gap in our knowledge which could be bridged by understanding how HN changes ‘over time’ in Alzheimer’s disease (i.e. longitudinal study). Indeed, evidence from the Dominantly Inherited Alzheimer Network study^35–37^ indicates that longitudinal analysis can provide a more accurate picture of disease progression. However, the lack of adequate techniques to study HN in the ‘living’ human brain limits the number of approaches that can be taken in research to address this gap effectively.

In the present study, we propose an *in vitro* parabiosis assay that models the impact of systemic environment on HN, which we have used as a proxy to investigate the changes in HN that occur with time. Using human HPCs and longitudinal serum samples from participants with MCI who either progressed to Alzheimer’s disease (MCI converters) or remained cognitively stable (MCI non-converters), we aimed to establish the role of the human systemic environment in disease progression *in vitro*. We also sought to determine whether our assay could be used as a prognostic biomarker to predict the likelihood of MCI to Alzheimer’s disease progression.

## Materials and methods

### Serum samples

Up to 161 serum samples were collected from 56 individuals initially diagnosed with MCI. Thirty-six individuals later developed dementia due to Alzheimer’s disease (denoted ‘MCI converters’, 2–5 yearly follow-up visits with cognitive assessment and blood collection). Eighteen did not progress either to Alzheimer’s disease or other disease, and they had transient memory problems while remaining cognitively stable over the period of at least 3 years from MCI diagnosis (denoted ‘MCI non-converters’, with up to six yearly follow-up visits with cognitive assessment and blood collection).

For serum preparation, blood was collected into Rapid Serum Tubes and allowed to stand for at least 30 min at room temperature (RT), then centrifuged at 2000*g* for 10 min at 4°C. The resulting serum was aliquoted into 2-ml flat-bottom screw-cap microcentrifuge tubes (0.5-ml serum/centrifuge tube) and stored at −80°C.

The serum samples were sourced from two independent cohorts. The first cohort is the EU AddNeuroMed Consortium, a multicentre European study^38^ with six participating medical centres: University of Kuopio (Finland), University of Perugia (Italy), Aristotle University of Thessaloniki (Greece), King's College London (UK), Medical University of Lodz (Poland) and University of Toulouse (France). Consensus diagnosis was made according to previously published criteria.^39,40^ Clinical diagnosis was confirmed during consecutive follow-up visits. Sixteen MCI non-converters and 34 MCI converters were recruited from this cohort (*n* = 50). The second cohort is the King’s Health Partners-Dementia Case Register, a UK clinic and population-based study.^38^ Diagnosis of probable Alzheimer’s disease was made according to the Diagnostic and Statistical Manual of Mental Disorders IV^40^ and NINCDS-ADRDA Alzheimer's criteria.^41^ MCI was diagnosed according to the criteria used by Petersen *et al.*^39^ Clinical diagnosis was confirmed during consecutive follow-up visits. Two MCI non-converter and four MCI converter participants were recruited from this cohort (*n* = 6). Informed written consent was obtained from all serum donors or their carers according to the Declaration of Helsinki (1991) and protocols and procedures were approved by the relevant Institutional Review Board at each collection site.

Longitudinal serum samples from the study participants were used to obtain the eight cellular readouts reported in this study, corresponding to each follow-up visit [proliferation phase average cell number, % Ki67^+^ cells, % cleaved Caspase 3 (CC3)^+^ cells; differentiation phase average cell number, % Ki67^+^ cells, % CC3^+^ cells, % doublecortin (DCX)^+^ cells, % microtubule-associated protein 2 (MAP2)^+^ cells]. A minimum of three serum samples, collected at annual assessment, was required for MCI non-converters; and a minimum of two samples, one before progression and one after progression, was required for MCI converters. Serum was collected at the time of cognitive assessments and the samples were stored at −80°C. The samples underwent one freeze–thaw cycle before performing experiments.

Baseline characteristics of serum donors are presented in Table 1, and changes in Mini-Mental State Examination (MMSE) scores over time are presented in Supplementary Fig. 1. All participants were age- (*P* = 0.320) and sex-matched (*P* = 0.129). MCI converters completed significantly fewer years of education compared to MCI non-converters (*P* = 0.002). They also scored significantly lower in MMSE (*P* = 0.031). There was no difference in the number of the apolipoprotein E (*APOE)* ε4 allele carriers between MCI converters and MCI non-converters (*P* = 0.625).

**Table 1:** Baseline characteristics of the study participants (*n* = 56)

Baseline characteristics	MCI converters	MCI non-converters
Gender, %, female	60.52	38.89
*APOE* ε4 status, %	50	44.44
Age at baseline, mean ± SD	76.02 ± 7.81	78.06 ± 5.08
MMSE at baseline, mean ± SD	26.78 ± 1.97	27.94 ± 1.43
Education in years, mean ± SD	8.95 ± 4.53	12.83 ± 3.09
Years from baseline until conversion or last assessment [range]	1.30 ± 0.69 [1–3.42]	3.36 ± 1.66 [0.92–5.83]
Comorbidities (objective and self-reported)		
ȃHypertension	12 (31.58%)	10 (55.56%)
ȃHeart attack ever	1 (2.63%)	4 (22.22%)
ȃAngina	5 (13.16%)	4 (22.22%)
ȃDepression	19 (50%)	9 (50%)
ȃArthritis	1 (2.63%)	3 (16.67%)
ȃAsthma	2 (5.26%)	1 (5.56%)
ȃCancer	5 (13.16%)	8 (44.44%)
ȃGlaucoma	2 (5.26%)	1 (5.55%)
ȃHypothyroidism	6 (15.79%)	8 (44.44%)
ȃInfections, allergies	5 (13.16%)	2 (11.11%)
ȃStroke/transient ischaemic attack	2 (5.26%)	2 (11.11%)
ȃDiabetes	7 (18.42%)	2 (11.11%)
Drug intake		
ȃAntidepressants	12 (31.58%)	3 (16.67%)
ȃNSAIDs (with aspirins for platelet control)	11 (28.95%)	8 (44.44%)
ȃAnalgesics	5 (13.16%)	5 (27.78%)
ȃAlzheimer’s disease drugs	11 (28.95%)	0 (0%)
ȃMetformin	4 (10.53%)	1 (5.55%)
ȃSleeping pills	3 (7.89%)	2 (11.11%)
ȃStatins	13 (34.21%)	8 (44.44%)
Lifestyle factors		
ȃExcessive alcohol intake	1 (2.63%)	3 (16.67%)
ȃSmoking (ever)	9 (23.68%)	5 (27.78%)
ȃSolitary living	16 (42.10%)	9 (50%)
ȃSupplement intake^a^	7 (18.42%)	5 (27.78%)

Comorbidities represent either history of disease or being presently affected. Drug intake means either having a history of medications or current intake.

Vitamins (including folic acid) and omega-3 fatty acids.

### Cell culture and serum treatment

All experiments were performed using the multipotent human hippocampal progenitor/stem cell line HPC0A07/03C (ReNeuron) derived from the first trimester female foetal hippocampal tissue following medical termination (in accordance with the UK and USA ethical and legal guidelines, and obtained from Advanced Bioscience Resources). HPC0A07/03C cells were conditionally immortalized by introducing *c-mycER^TAM^* transgene that enables them to proliferate indefinitely in the presence of epidermal growth factor (EGF), basic fibroblast growth factor (bFGF) and 4-hydroxy-tamoxifen (4-OHT).^42^ Removal of these three factors induces spontaneous differentiation into neurons, astrocytes or oligodendrocytes.^16,43,44^ Cell passage number used in this study ranged from 15 to 24.

**Figure 1:**
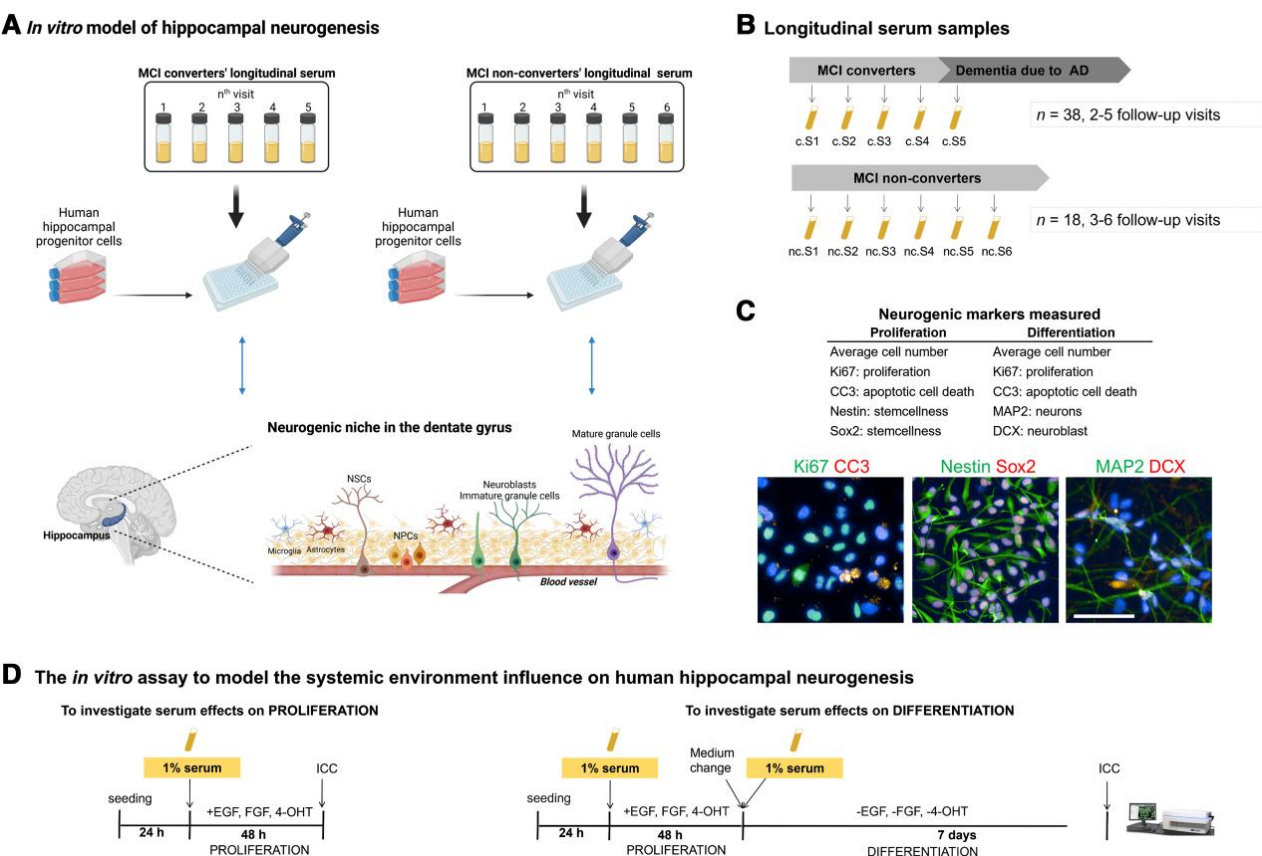
**Outline of the experimental design and sample collection**. (**A**) HN is regulated by a complex microenvironment, composed of blood vessels and various cell types such as hippocampal neural stem cells (NSCs), neural progenitor cells (NPCs), neuroblasts, immature/mature granule cells (i.e. neurons), microglia and astrocytes (i.e. neurogenic niche). Blood-derived factors, delivered to the niche by its rich vasculature, play a fundamental role in modulating HN. We aimed to model the role of systemic environment on the hippocampal neurogenic process during Alzheimer’s disease progression, by treating a human HPC line with 1% longitudinal serum at different stages of HN (i.e. proliferation and differentiation of HPCs). (**B**) Longitudinal serum samples were collected during annual follow-up visits from 56 participants diagnosed with MCI at baseline (*n* = 38 converted to Alzheimer’s disease, *n* = 18 remained cognitively stable). A total of 338 samples were analysed. For each sample, three biological replicates (cells of three different passage numbers) were used and for each biological replicate, there were technical triplicates. (**C**) Neurogenic markers measured in the proliferation and differentiation phases of the assay are outlined (*top*) and representative images of cells positive for Ki67, CC3, Nestin, Sox2, MAP2 and DCX are shown (*bottom*). Scale bar = 100 μm. (**D**) An overview of the proliferation and differentiation phases in the assay. HPC0A07/03C cell line was treated with 1% serum samples from MCI converters and non-converters collected at sequential follow-up visits. Proliferation medium included EGF, bFGF and 4-OHT. Differentiation medium lacked these factors. To analyse serum effects on proliferation, 24 h after seeding, medium was replaced with proliferation medium supplemented with 1% serum. Cells were fixed 48 h later and subjected to ICC. To analyse the effects of serum on differentiation, at the end of proliferation phase, medium was replaced with differentiation medium supplemented with 1% serum. Cells were fixed 7 days later and subjected to ICC. c = converters; nc = non-converters. Panel **A** was created with BioRender.com.

Cells were treated with 1% serum 24 h post-seeding for the proliferation assay; and for the differentiation assay cells were treated with serum 24 h post-seeding in proliferation medium, and one more time 3 days post-seeding in differentiation medium (Fig. 1). See Anacker *et al.*^16^ and Supplementary Table 1 for cell culture medium composition and de Lucia *et al.*^45^ for information on how the optimal serum concentration was determined. Control conditions consisted of either proliferation or differentiation medium supplemented with 1% Gibco^™^ PenStrep (ThermoFisher, #15140122). For each experiment, three biological replicates (i.e. cells of three different passage numbers) were used; and for each biological replicate, there were technical triplicates. The coefficient of variation for each marker was below 20%, apart from CC3 (below 30%), calculated across different plates and batches of experiment. Further details on the methods can be found in the Supplemental Material.

### Immunocytochemistry

All experiments were performed in NUNC™ 96-well plates (ThermoFisher, #167008). Cells were fixed in 4% paraformaldehyde (VWR, #43368.9 M) after 48 h of serum treatment for the proliferation and 7 days of treatment for the differentiation phase of the assay, respectively.

Briefly, cells were washed once with 37°C PBS and fixed in 4% paraformaldehyde at RT for 20 min (50 µl/well), then they were washed twice with PBS for storage at 4°C prior to immunocytochemistry (ICC). On the day of ICC, cells were first blocked in ‘5% normal donkey serum + 0.3% Triton X-100’ in PBS (i.e. blocking solution) at RT for 1 h (50 µl/well). On removal of blocking solution, cells were incubated with primary antibodies diluted, overnight at 4°C (30 µl/well). Cells were then washed with PBS (100 µl/well) twice and incubated with secondary antibodies (1:500) at RT for 2 h (30 µl/well) covered from light. On removal of secondary antibodies, cells were washed with PBS (100 µl/well) twice and incubated with 4′,6-diamidino-2-phenylindole (DAPI) nuclear stain (Sigma Aldrich, #D9542) at RT for 5 min (50 µl/well). Finally, cells were washed with PBS (100 µl/well) twice and stored at 4°C with 0.05% sodium azide in PBS (200 µl/well) before imaging.

All primary and secondary antibody solutions were made in blocking solution (as described before). Mouse monoclonal anti-Ki67 (Cell Signaling, #9449, 1:800) was used to assess proliferation (i.e. HPCs in active phases of the cell cycle such as G1, S, G2 and mitosis); rabbit monoclonal anti-CC3 (Cell Signaling, #9664, 1:500) to assess apoptotic cell death; mouse monoclonal anti-Nestin clone 10C2 (Sigma Aldrich, #MAB5326, 1:1000) and rabbit polyclonal anti-Sox2 (SRY-Box Transcription Factor 2) (Sigma Aldrich, #AB5603, 1:1000) to assess neural stemcellness; rabbit polyclonal anti-DCX (Abcam, #ab18723, 1:500) for neuroblasts and mouse monoclonal anti-MAP2 (Abcam, #ab11267, 1:500) for mature neurons. Secondary antibodies were conjugated with either Alexa 488 (Thermo Fisher Scientific, #A21202) or Alexa 555 (Thermo Fisher Scientific, #A31572) fluorophores. Nuclei were counterstained with DAPI (Sigma Aldrich, #D9542).

### High-content imaging

Semi-automated quantification of cellular phenotypes was performed using the CellInsight™ CX5 High-Content Screening Platform (ThermoFisher). The ‘Cell Health Profiling’ application was used to detect the nucleus (DAPI) and to quantify neurogenic markers expressed in the nucleus (Ki67), and the cell body/dendrites (CC3, DCX and MAP2) (High-Content Screening Studio™ Cell Analysis Software, ThermoFisher). Based on the values from positive and negative staining controls, thresholds were set for average intensity within the target regions of interest (e.g. nuclear or cell body). Any cell with an average intensity bigger than the threshold was deemed positive for a given neurogenic marker. Fifteen fields were scanned per well of a 96-well plate. A representative protocol used for cellular phenotyping is shown in Supplementary Fig. 2.

### Statistics

All statistical analyses were performed using Prism v.5.0 (GraphPad), STATA 13 or R. For univariate analyses, two-tailed paired *t*-test, one- and two-way ANOVA, with *post hoc* comparisons test (Bonferroni method) were used. The chi-square test was carried out to test for differences in categorical outcomes such as sex and *APOE* ε4 status. Complete case analyses were performed in this study. Source data are provided in Supplementary Table 2.

### Linear mixed-effects regression

Owing to the longitudinal aspect of our dataset, we used linear mixed-effects regression models for repeated measures as they enable inclusion of varying numbers of assessment information available for each individual and do not require equal time intervals between the follow-up visits. Random intercept and random slope models were fitted with restricted maximum likelihood as the method of estimation. Each serum donor was assigned an ID to specify random effects in the models. Classification to MCI converters or non-converters was dichotomous (MCI converters were assigned 1, non-converters 0). The age of the individuals was centred at the cohort median (77 years) to aid interpretation of the models. For MCI converters, time to conversion was measured in years, which indicated the time it took to Alzheimer’s disease progression. For non-converters, time to last visit was measured in years. Time before conversion (or last visit) was assigned negative values and time after conversion positive values. *APOE* ε4 status was dichotomized, i.e. carriers with at least one *APOE* ε4 allele were assigned 1, carriers of other *APOE* alleles were assigned 0. Education was entered in the models either as years of education or as a dichotomized value (high ≥ 10.5 years assigned 1, low <10.5 years assigned 0). The 95% confidence intervals (CIs) and *P*-values for the explanatory variables in each model were calculated using the Wald *t*-distribution.

Given that many individual characteristics or comorbidities might affect HN and/or Alzheimer’s disease risk, among the potential explanatory variables considered in the models were: Alzheimer’s disease risk factors [gender, *APOE* ε4 status, age centred around median and time to conversion (or time from last visit for MCI non-converters)], education level, solitary living and MMSE score (baseline MMSE, MMSE score change/year); comorbidities (diabetes, arthritis, hypertension, hypothyroidism, depression, cancer, stroke, angina, infections and allergies); drug intake (antidepressants, statins, nonsteroidal anti-inflammatory drugs); dietary supplements (vitamins, omega-3 fatty acids) and lifestyle related factors (alcoholism, smoking). In addition, we also tested some biologically plausible interactions of different explanatory variables.

Our approach to building the linear mixed-effects model was to systematically compare the full model to other models that were the same except for one term missing. The comparison was done using a likelihood-ratio test, and the test statistic χ^2^, degrees of freedom and *P*-value were reported for the missing term. A *P*-value of <0.05 was considered to indicate that the missing term contributed significantly to the model fit. We only included variables that were significant when included in the model. All mixed-effects regression models were assessed using Akaike information criterion, likelihood-ratio test and deviance.

### Stepwise logistic regression and internal validation

Stepwise logistic regression analysis was carried out to assess the effect of selected predictors on probability of progression to Alzheimer’s disease by 3.5-year follow-up from baseline. It was preceded by analysis of multicollinearity. Area under the curve (AUC) under the receiver operating characteristic (ROC) curve was calculated to determine the classification accuracy of selected variables in predicting progression to Alzheimer’s disease.

The ‘e1071’ package in R was used to train and test the machine learning classifiers based upon support-vector machines (SVM) classifier using the radial basis function kernel. ROC curves were drawn using the ‘ROCR’ package in R. Performance of the classifier was assessed using 1000 repeats of 5-fold cross-validation.

### Proteomic quantification

Here, 3620 unique proteins or 4006 different protein epitopes were quantified using the SomaScan assay (SomaLogic Inc.) from 150 µl of baseline serum samples from 38 MCI converters and 18 MCI non-converters. The SomaScan assay is an aptamer-based technology that uses protein-capture SOMAmers (Slow Off-rate Modified Aptamer) to quantify proteins in a biofluid. SOMAmers are chemically modified oligonucleotides with specific affinity to their protein targets, developed by SELEX (described in detail at www.somalogic.com). The identities of all proteins quantified are listed in Supplementary Table 3. The normalized and calibrated signal for each SOMAmer reflects the relative amount of each cognate protein present in the original sample. Quantifications are reported in relative fluorescence units and all data were first log_10_ transformed before analysis.

### Analysis of SomaScan data

A Wilcox test with false discovery rate multiple correction was used to identify proteins that were differentially expressed. Machine learning using least absolute shrinkage and selection operator feature selection and SVM for prediction was performed to identify the optimal number of multivariate proteins to differentiate MCI converters from non-converters. Samples were divided into non-intersecting subsets. Training and testing were performed on these following standard 10-times cross-validation. Briefly, the data is randomly partitioned into 10 parts; each model is built using nine of the parts as a training set and one part as the test set. The 10 models are averaged to create a single model balanced for randomness.

### Ingenuity pathway analysis

IPA (ingenuity pathway analysis, IPA^®^, Qiagen) generated a list of canonical pathways and networks for proteins within detection limit of the SomaScan. Only proteins differentially expressed with *P* < 0.05 were considered for analysis.

### Data availability

Further information on resources and reagents should be directed to and will be fulfilled by the lead contact, S.T. This study did not generate new unique reagents and codes for data analysis. The HPC0A07/03C cell line (ReNeuron) and further information on reagents needed for culturing this cell line are available from the lead contact on request. Source data for all figures and tables are provided within this study as supplemental information (available online). Any additional information required to reanalyse the data reported in this paper is available from the lead contact upon request. The transparent reporting of a multivariable prediction model for individual prognosis or diagnosis guidelines were used.^46^ All supporting data and associated links can be found in the Supplementary material.

## Results

### Longitudinal changes in neurogenic readouts characterizing serum from MCI converters

First, using the longitudinal serum samples from MCI converters only, we modelled the relationship between time to conversion in years, as an explanatory variable, and each readout readout from our assay, as a response variable, using linear mixed-effects regression. We used time to conversion in years as the explanatory variable because the effect of age itself in years on HN level was not significant (*P* > 0.05). The value 0 was assigned to the time of Alzheimer’s disease diagnosis, which equals the time point when the last serum sample was collected for each MCI converter. Other samples collected before that time were assigned negative values in years (i.e. serum taken 1 year before conversion was assigned −1).

**Figure 2:**
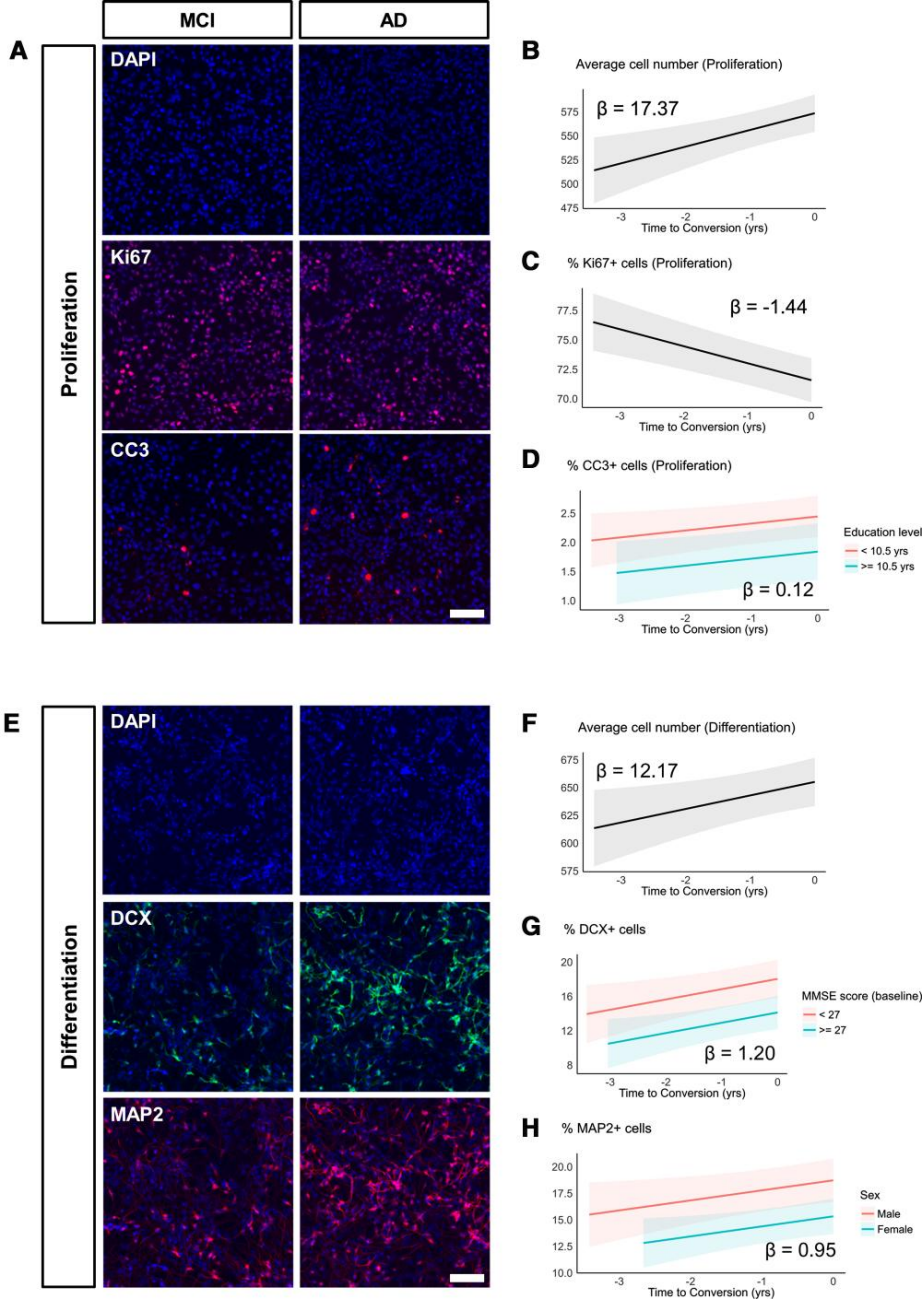
**Exposure to 1% serum from MCI converters leads to decreased proliferation, increased cell death and increased neurogenesis**. (**A**) Representative images of proliferation phase cells treated with serum from the same individual. Left (MCI panel): serum sample from 1 year before conversion. *Right* (AD panel): serum sample taken at the time of conversion to Alzheimer’s disease (AD). Nuclei are stained with DAPI. Ki67 and CC3 were used to label proliferating and apoptotic cells, respectively. (**B**–**D**) Modelled trajectories (with 95% CIs) of linear mixed-effects regression models fitted to the proliferation phase data. Time of conversion to Alzheimer’s disease was assigned 0, and the number of years before conversion were assigned negative values (i.e. 1 year before conversion is −1). Longitudinal serum samples from MCI converters increased average cell number (**B**), decreased proliferation (% Ki67^+^) (**C**) and increased apoptotic cell death (% CC3^+^) (**D**). Slopes (β coefficient estimates) are indicated within the plots. (**E**) Representative images of differentiation phase cells treated with serum from the same individual. *Left* (MCI panel): serum sample from 1 year before conversion. *Right* (AD panel): serum sample taken at the time of conversion to AD. Nuclei are stained with DAPI. DCX and MAP2 were used to label neuroblasts and mature neurons, respectively. (**F**–**H**) Modelled trajectories (with 95% CIs) of linear mixed-effects regression models fitted to the differentiation phase data. Time of conversion to Alzheimer’s disease was assigned 0, and the number of years before conversion were assigned negative values (i.e. 1 year before conversion is −1). Longitudinal serum samples from MCI converters increased average cell number (**F**), neuroblasts (% DCX^+^) (**G**) and mature neurons (% MAP2^+^) (**H**). Slopes (β coefficient estimates) are indicated within the plots. Scale bar = 100 μm.

When random intercept models were fitted to the proliferation phase data (Fig. 2A–D, Supplementary Fig. 3 and Supplementary Table 4), the effects of time to conversion on average cell number were significantly positive [beta = 17.37, 95% CI: 6.87 to 27.87, *t*(81) = 3.29, *P* = 0.001]. This was not related to an increase in proliferation itself (% Ki67 + cells) because the effects of time of conversion were significantly negative over the same period [beta = −1.44, 95% CI: −2.03 to −0.86, *t*(81) = −4.94, *P* < 0.001]. In addition, the effects of time to conversion on apoptotic cell death (% CC3^+^ cells) were significantly positive [beta = 0.12, 95% CI: 0.01 to 0.23, *t*(80) = 2.19, *P* = 0.031], while the effect of education level (dichotomized at 10.5 years) was significantly negative on apoptotic cell death [beta = −0.60, 95% CI: −1.20 to −0.01, *t*(80) = −2.03, *P* = 0.046].

When the models were fitted to the differentiation phase data (Fig. 2E–H, Supplementary Fig. 2 and Supplementary Table 5), an increase with time to conversion was observed for average cell number [beta = 12.17, 95% CI: 2.24 to 22.11, *t*(81) = 2.44, *P* = 0.017], number of neuroblasts [% DCX^+^ cells, beta = 1.20, 95% CI: 0.27 to 2.12, *t*(79) = 2.58, *P* = 0.012] and number of mature neurons [% MAP2^+^ cells, beta = 0.95, 95% CI: 0.10 to 1.79, *t*(80) = 2.22, *P* = 0.029]. For the number of neuroblasts, baseline MMSE scores (dichotomized at 27) were also found to be a significant explanatory variable, where higher MMSE scores at baseline had a significantly negative effect on DCX^+^ cells overall [beta = −3.91, 95% CI: −6.75 to −1.08, *t*(79) = −2.75, *P* = 0.007]. Similarly, sex (female assigned 1) was a significantly negative explanatory variable for the number of mature neurons [beta = −3.39, 95% CI: −5.90 to −0.89, *t*(80) = −2.70, *P* = 0.009].

We did not detect any significant effects of time to conversion on apoptotic cell death in the differentiation phase data [beta = 0.16, 95% CI: −0.09 to 0.40, *t*(81) = 1.28, *P* = 0.203], and variables such as *APOE* ε4 status and comorbidities (as listed in Table 1) did not have significant explanatory values when included in the mixed-effects models for both proliferation and differentiation phase datasets. No significant interactions between predictors were determined in the models we tested (Supplementary Fig. 5).

### Serum from MCI converters differentially impacts neurogenic readouts compared to non-converters

Next, we asked whether serum from MCI converters and non-converters can differentially impact the trajectories of HN. We used the variable ‘MCI to Alzheimer’s disease progression’ to denote whether the participant progressed to Alzheimer’s disease or not (converters assigned the value 1).

**Figure 3:**
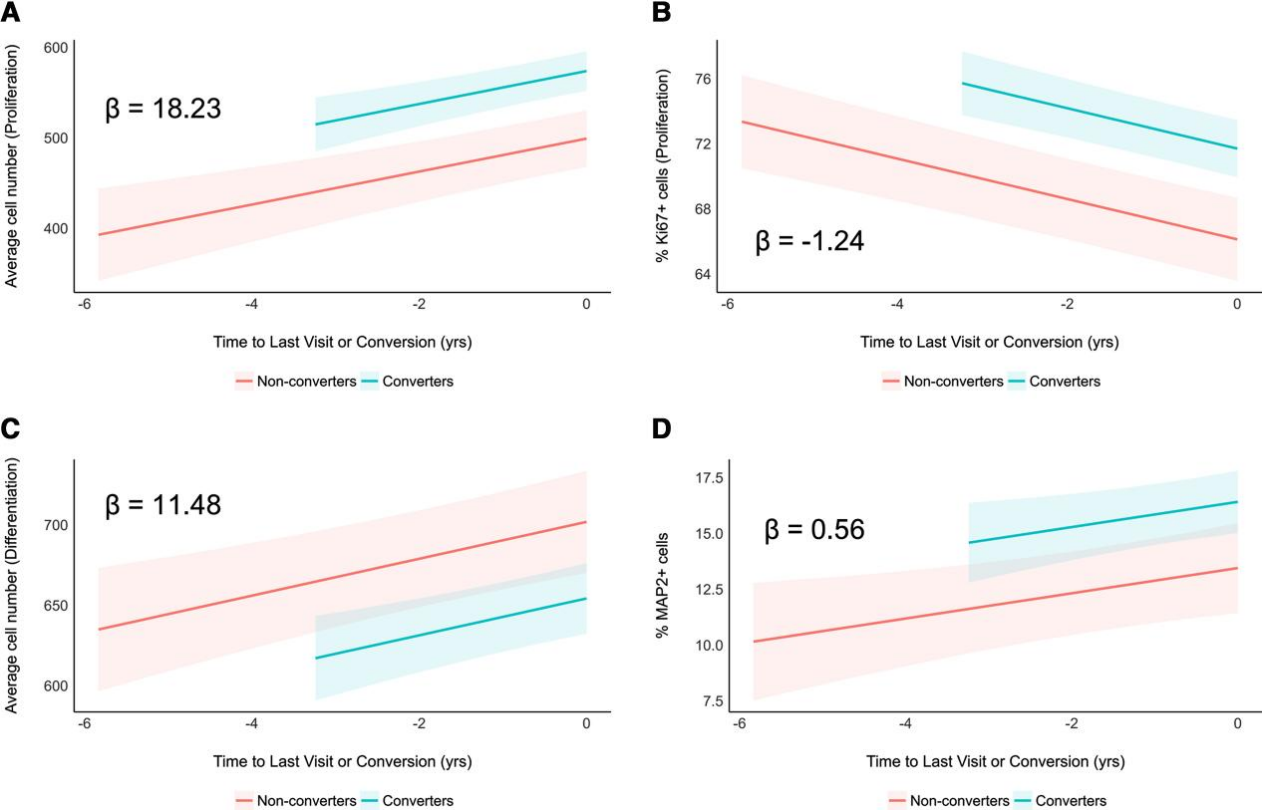
**Exposure to 1% serum from MCI converters leads to differential changes in average cell number, proliferation and neuronal differentiation compared to non-converters**. (**A** and **B**) Modelled trajectories (with 95% CIs) of linear mixed-effects regression models fitted to the proliferation phase data. Time to last visit (for non-converters) and time of conversion to Alzheimer’s disease (for converters) was assigned 0, and the number of years before that were assigned negative values (i.e. 1 year before conversion is −1). Longitudinal serum samples from MCI converters (turquoise) predicted overall higher average cell number (**A**) and proliferation (% Ki67^+^) (**B**) compared to non-converters (red). Slopes (β coefficient estimates) are indicated within the plots. (**C** and **D**) Modelled trajectories (with 95% CIs) of linear mixed-effects regression models fitted to the differentiation phase data. Time to last visit (for non-converters) and time of conversion to Alzheimer’s disease (for converters) was assigned 0, and the number of years before that were assigned negative values (i.e. 1 year before conversion is −1). Longitudinal serum samples from MCI converters (turquoise) predicted overall lower average cell number (**C**) and higher neuronal differentiation (% MAP2^+^) (**D**) compared to non-converters (red). Slopes (β coefficient estimates) are indicated within the plots.

Fitting the linear mixed-effects models on the proliferation phase data (Fig. 3A and B, Supplementary Fig. 3 and Supplementary Table 6), we observed significantly positive effects of both time to last visit or conversion [beta = 18.23, 95% CI: 10.81 to 25.65, *t*(154) = 4.85, *P* < 0.001] and MCI to Alzheimer’s disease progression [beta = 74.71, 95% CI: 36.96 to 112.46, *t*(154) = 3.91, *P* < 0.001] on average cell number. On the other hand, their effects were significantly negative on proliferation (% Ki67^+^ cells) [time to last visit or conversion: beta = −1.24, 95% CI: −1.59 to −0.89, *t*(155) = −7.03, *P* < 0.001; MCI to Alzheimer’s disease progression: beta = 5.58, 95% CI: 2.53 to 8.63, *t*(155) = 3.61, *P* < 0.001].

During the differentiation stage of the assay (Fig. 3C and D, Supplementary Fig. 4 and Supplementary Table 7), we observed significantly positive effects of time to last visit or conversion [beta = 11.48, 95% CI: 5.65 to 17.32, *t*(156) = 3.89, *P* < 0.001] and significantly negative effects of MCI to Alzheimer’s disease progression [beta = −47.64, 95% CI: −85.13 to −10.15, *t*(156) = −2.51, *P* = 0.013] on average cell number. On the other hand, their effects were significantly positive on the number of mature neurons (% MAP2^+^ cells) [time to last visit or conversion: beta = 0.56, 95% CI: 0.11 to 1.02, *t*(153) = 2.47, *P* = 0.015; MCI to Alzheimer’s disease progression: beta = 2.96, 95% CI: 0.62 to 5.31, *t*(153) = 2.50, *P* = 0.014]. We did not detect any significant effects time to last visit or conversion on apoptotic cell death in the differentiation phase data [beta = 0.02, 95% CI: −0.09 to 0.14, *t*(153) = 0.41, *P* = 0.680], but effects of MCI to Alzheimer’s disease progression were significantly positive [beta = 1.62, 95% CI: 0.89 to 2.36, *t*(153) = 4.36, *P* < 0.001] (Supplementary Fig. 6). Taken together, our data show that, compared to non-converters, converters can be characterized with higher average cell number and proliferation during the proliferation phase of the assay, and then with lower average cell number and more mature neurons during the differentiation phase of the assay.

### Baseline neurogenic readouts and education can predict MCI to Alzheimer’s disease progression

We next examined whether the baseline neurogenic readouts from the assay combined with some of the baseline participant characteristics could predict progression from MCI to Alzheimer’s disease. Using stepwise logistic regression, the best predictors of progression from MCI to clinical Alzheimer’s disease were: education in years, average cell number during proliferation phase, % Ki67^+^ cells during proliferation phase and % CC3^+^ cells during differentiation phase of the assay (Table 2). The fit of the logistic regression model was confirmed using Hosmer–Lemeshow goodness of fit (*P* = 0.324) and Stata linktest, demonstrating no specification errors (_hat = 0.001, _hatsq = 0.110). We observed no significant effects of education (both in years and dichotomized at 10.5 years) on average cell number during proliferation phase, percentage Ki67^+^ cells during proliferation phase, and percentage CC3^+^ cells during differentiation phase of the assay (Supplementary Fig. 7).

**Table 2:** Predictors of progression to Alzheimer’s disease from stepwise logistic regression analysis

Predictors	Odds ratios	95% CI	*P*	
**Model with all four predictors**				
(Intercept)	0	0.00–0.00	0.012	
Education (years)	0.72	0.49–0.94	0.039	
Average cell number (prol.)	1.03	1.01–1.05	0.001	
% Ki67^+^ cells (prol.)	1.35	1.07–1.92	0.037	
% CC3^+^ cells (diff.)	3.49	1.42–11.85	0.016	
**Predictors**	**Odds ratios**	**95% CI**	***P***	**AUC**
**Four models with one predictor each**				
Education (years)	0.79	0.66–0.92	0.004	0.756
Average cell number (prol.)	1.02	1.01–1.02	<0.001	0.802
% Ki67^+^ cells (prol.)	1.04	0.94–1.16	0.399	0.573
% CC3^+^ cells (diff.)	2.67	1.48–5.72	0.004	0.782

*Top*: The full logistic regression model including all four predictors: years of education, average cell number during proliferation, proliferation marker (Ki67) during proliferation and apoptosis marker (CC3) during differentiation stages of the assay. *Bottom*: Four logistic regression models with each predictor being the sole predictor of progression to Alzheimer’s disease. Odds ratios, 95% CI and *P*-values are shown. prol = proliferation assay; diff = differentiation assay.

**Figure 4:**
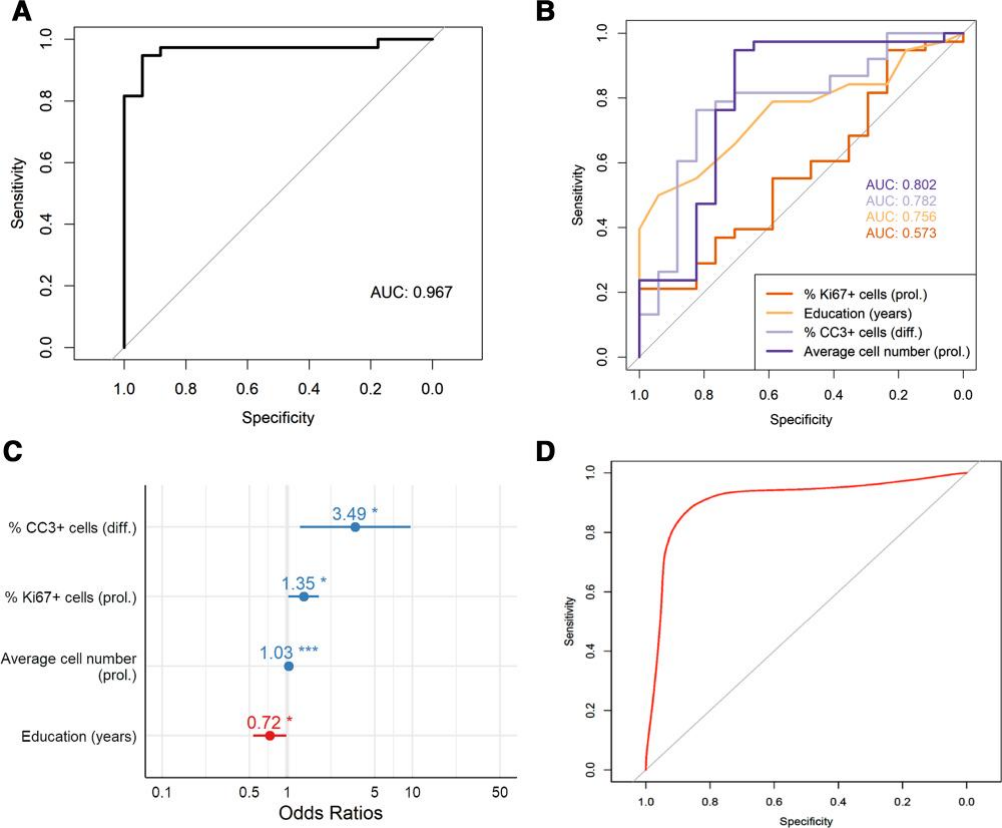
**Average cell number and Ki67 during proliferation, and CC3 during differentiation, combined with education in years can predict progression from MCI to Alzheimer’s disease**. (**A**) ROC curve for the logistic regression model predicting progression from MCI to Alzheimer’s disease. AUC for the model, an indicator of the discriminative performance, is 0.967. Sensitivity = 92.1%, specificity = 94.1%, positive predictive value = 97.2% and negative predictive value = 84.2%. (**B**) ROC curves for each four individual predictors included in the full logistic regression model. (**C**) Odds ratios for the four predictors. Blue and red indicate >1 and <1, respectively. *P < 0.05. ***P < 0.001. (**D**) ROC curve for the cross-validated logistic regression model predicting progression to Alzheimer’s disease. Internal validation of the model was done with repeated *k*-fold cross-validation (*k* = 5, 1000 repeats) using SMVs (radial basis function kernel). AUC = 0.93, sensitivity 90.3% and specificity 79.0%.

To assess the predictors’ ability to accurately classify converters and non-converters, area under the ROC curve was calculated (Fig. 4A). The value of the full logistic regression model, 0.967, was higher than that of other models built on each predictor alone (Fig. 4B and Table 2). We also found that the odds of converting to Alzheimer’s disease decreased by factor 0.72 with each additional year of education, whereas it increased by a factor of 3.49 with each additional percentage point of apoptotic cell death during the differentiation phase of the assay (Fig. 4C).

Since our sample size was limited (*n* = 56) and we did not have access to a separate longitudinal validation cohort in this study, a machine learning-based internal validation of the model was performed. Repeated *k*-fold cross-validation (*k* = 5, 1000 repeats) was carried out using a SVM classifier (radial basis function kernel), in which 20% of the data were used for each round of repeated testing. We found that the classifier using the three chosen neurogenic readouts as predictors achieved an AUC of 0.93, with 90.3% sensitivity and 79.0% specificity (Fig. 4D).

### Proteomic analysis of baseline serum from MCI converters and non-converters

**Figure 5:**
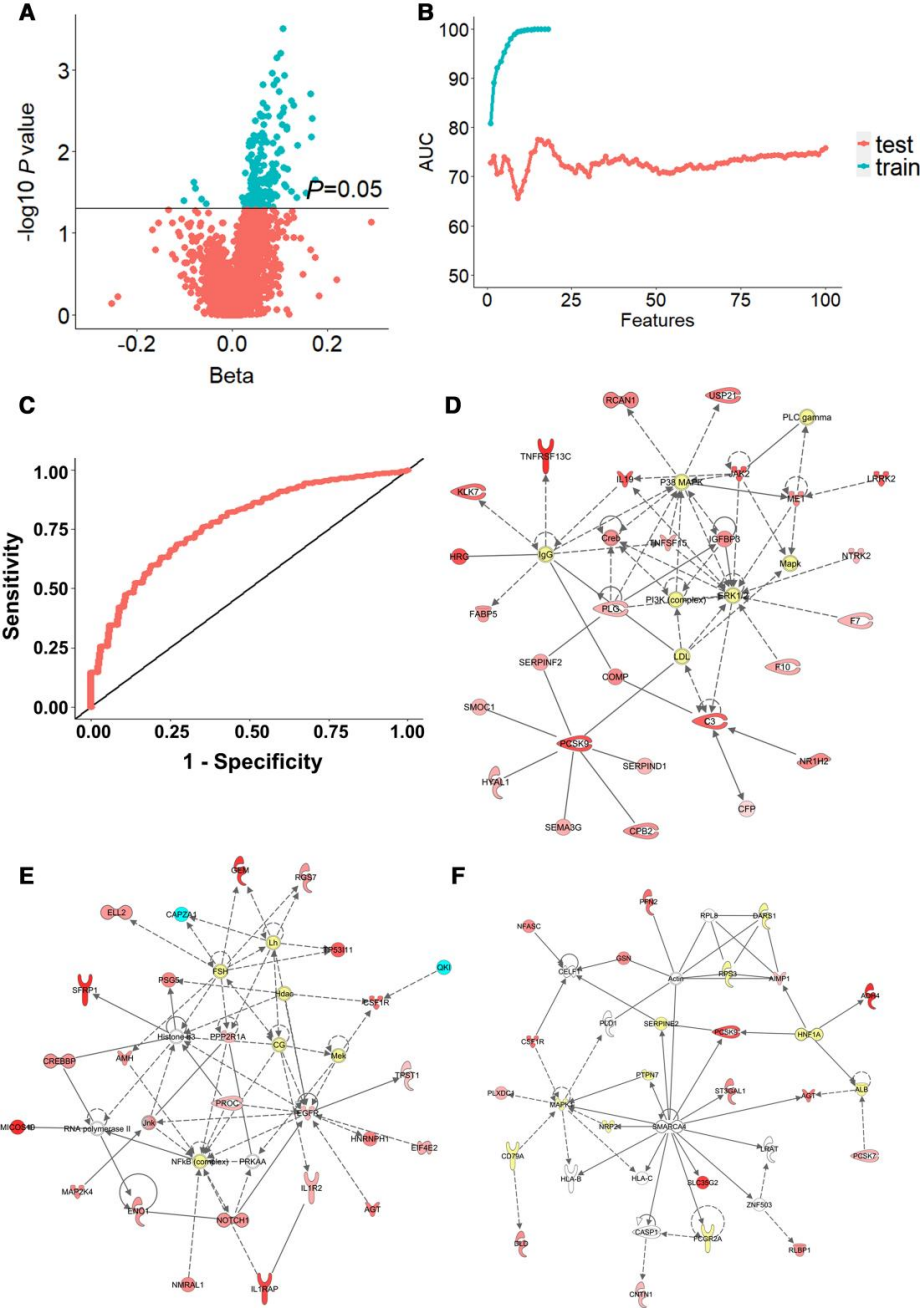
**Analysis of serum proteins differentially expressed in MCI converters compared to non-converters**. (**A**) Volcano plot of proteins significantly increased in MCI converters compared to non-converters. Turquoise dots represent proteins whose *P*-value was below the significance threshold of *P* < 0.05 (*n* = 205). (**B**) The optimal number of multivariate proteins to differentiate MCI converters from non-converters was calculated by machines learning. The plot shows AUCs of the training set as features are added to the model, and AUCs of the testing set during cross-validation. The least absolute shrinkage and selection operator feature selection with SVM for prediction were used. The number of proteins identified with this procedure was 15. (**C**) ROC curve of the test model. The maximum AUC (0.77) was achieved using the following 15 identified proteins: Q9UHD0, Q9UK55, Q9NPH3, Q96PU8, Q8N474, Q8TBE7, Q9NTK1, Q6UWD8, O14548, P43251, P19876.P19875, Q8NBP7, P52907, P00797 and Q9Y5Q6 (UniProt ID). (**D**–**F**) The three top scoring networks analysed by IPA using the 205 differentially expressed proteins in MCI converters. Proteins are represented by nodes: upregulated in red and downregulated in turquoise. Additional interacting molecules not included in the SomaScan are marked in white. Each network is displayed as a series of nodes (proteins) and edges (i.e. lines corresponding to biological relationships between nodes). Solid and dotted lines indicate direct and indirect interactions, respectively. (**D**) ‘Hematological System Development and Function, Organismal Functions, Organismal Injury and Abnormalities’ (score 48; includes 27 focus molecules from the SomaScan panel). (**E**) ‘Cell Death and Survival, Embryonic Development, Organismal Development’ (score 43; includes 25 focus molecules from the SomaScan panel). (**F**) ‘Cell-to-Cell Signaling and Interaction, Cellular Function and Maintenance, Inflammatory Response’ (score 21; includes 15 focus molecules from the SomaScan panel).

To explore whether we can achieve a similar prognostic accuracy using a different modality, we performed a proteomic analysis on all baseline serum samples using SomaScan® (SomaLogic). The serum levels of 205 proteins (Fig. 5A and Supplementary Table 8) were found to be significantly differentially expressed between MCI converters and non-converters. However, none of these passed the false discovery rate correction. Among the differentially expressed proteins, there were proteins involved either in the neurogenic process (e.g. GDF11) or in Alzheimer’s disease (e.g. LRRK2, RCAN1, NTRK2), or in both neurogenesis and Alzheimer’s disease (e.g. CREBBP, SFRP1, IL1RAP). We then performed machine learning-based repeated *k*-fold cross-validation (*k* = 10) to find the minimal signal that differentiated between MCI converters and non-converters. The AUC in training and testing sets for different number of input features is shown in Fig. 5B. A panel of 15 proteins achieved the highest predictive value AUC of 0.77 to distinguish between serum samples from MCI converters and non-converters (Fig. 5C and Supplementary Table 9).

We then aimed to gain further insights into molecular pathways and networks by which proteins in the serum might regulate hippocampal stem cell fate and progression to Alzheimer’s disease. Canonical pathway analysis and Network analysis on IPA software (Qiagen) was used to determine any pathways that the differentially expressed proteins might constitute. Some of the canonical pathways identified in the analysis include: ‘Coagulation system’ (*P* = 0.000192, ratio 7/26), ‘Acute phase response signalling’ (*P* = 0.00345, ratio 12/100), ‘Extrinsic prothrombin activation pathway’ (*P* = 0.0111, ratio 3/10), ‘FXR/RXR activation’ (*P* = 0.0146, ratio 7/53), ‘Notch signaling’ (*P* = 0.0237, ratio 3/13), ‘Superpathway of methionine degradation’ (*P* = 0.0321, ratio 2/6) and ‘Wnt/β-catenin Signaling’ (*P* = 0.0353, ratio 6/50) (Supplementary Table 10). The three top networks identified in the analysis were: ‘Hematological System Development and Function, Organismal Functions, Organismal Injury and Abnormalities’ (Fig. 5D and Supplementary Table 11), ‘Cell Death and Survival, Embryonic Development, Organismal Development’ (Fig. 5E and Supplementary Table 11) and ‘Cell-to-Cell Signaling and Interaction, Cellular Function and Maintenance, Inflammatory Response’ (Fig. 5F and Supplementary Table 11).

## Discussion

This study used an *in vitro* parabiosis assay where a human HPC line was exposed to longitudinal samples of human serum. Our approach to modelling the effects of systemic milieu on HN can serve as a proxy of *in vivo* HN, as HPCs are allowed to react to a given systemic environment (such as serum) sampled at different time points. We demonstrate that the baseline data generated from the assay were able to predict progression from MCI to Alzheimer’s disease up to 3.5 years before clinical diagnosis, providing an opportunity to understand the temporal changes of HN at the early stages of Alzheimer’s disease progression.

We report an increase in neurogenesis induced by serum obtained closer to the time of MCI to Alzheimer’s disease progression. While previous human autopsy studies showed dysregulation of HN in Alzheimer’s disease, it has been debated whether HN is increased,^32^ decreased^23,24,47^ or unchanged.^48^ As most of these studies describe HN at the ‘later’ stages of Alzheimer’s disease, it is difficult to extrapolate their results to ‘early’ stages of Alzheimer’s disease. We note that our *in vitro* measures are only potential proxy of *in vivo* HN, and the systemic effect on the neurogenic process *in vivo* is more likely to be visible later than what we observe *in vitro*. Nevertheless, our data showing increased proliferation in MCI converters during the differentiation stage of the assay are in line with a recent rodent study that investigated HN in ‘prodromal’ Alzheimer’s disease, where proliferation of DCX^+^ neuroblasts in the hippocampus was significantly and specifically ‘elevated’ during the pre-plaque stage in the APP-PS1 mouse model.^25^ Intriguingly, we observed an increased average cell number and a decreased percentage of proliferating cells with each consecutive visit. This may be due to increased proliferation at an earlier time point, followed by contact inhibition of proliferation at 48 h after serum treatment,^49^ suggesting that it might be informative to include an earlier time point in the assay. In addition, we observed an increased apoptosis that could be related to the depletion of nutrients and increase in metabolites such as lactate as cells become overconfluent.^50–52^

It is not clear whether increased HN plays a compensatory role by providing cognitive resilience or contributes to ongoing pathology in Alzheimer’s disease. For example, increased neurogenesis was associated with behavioural recovery in a mouse model of selective neuronal loss in the hippocampus (CaM/Tet-DTA), although this effect was only pronounced in young mice (6 months old)^53^ and not in old mice (14 months old).^54^ This suggests that increased HN at the later stages of Alzheimer’s disease might be insufficient for cognitive recovery. In contrast to the rescuing effects of HN, several functional studies have shown that increased HN can interfere with the retrieval of old memories,^55–57^ while ablation of neurogenesis can improve hippocampus-dependent working memory by reducing interference.^58,59^ While the exact role of ‘increased’ HN in Alzheimer’s disease still remains to be determined, we attempt to make a cautious summary of the findings from our study, in which increased neurogenesis could be a compensatory mechanism in response to the ageing/neurodegenerative systemic milieu, but it may not be a functionally restorative one that could halt cognitive decline altogether.

The prediction model in this study was able to differentiate MCI converters from non-converters using a subset of the baseline data from the assay and years of education. We believe that education attainment serves as a proxy of ‘lifestyle’, where it may affect the choice of occupation, socioeconomic status and the degree of exposure to Alzheimer’s disease risk factors throughout life. While our study supports previous findings on the association between lower education and higher Alzheimer’s disease risk,^60^ we note that various lifestyle factors that may provide even better prediction of HN were not directly examined in this study. This includes (but not limited to) social/cognitive engagement, physical activity and diet. For those that were available for this study, we report no significant effects on the trajectories of HN modelled in our assay, except for a few demographic characteristics such as education level dichotomized at 10.5 years, baseline MMSE scores and sex. In addition, we report no significant effect of *APOE* ε4 status, adding to the existing literature on the ‘debatable’ role of ε4 in the hippocampus.^61–63^

The baseline neurogenic readouts from our assay were able to predict progression into clinical Alzheimer’s disease with higher accuracy than a panel of 15 serum proteins that were identified from proteomic analysis. This could be because neurogenic readouts represent the effect of ‘all components’ in the serum (i.e. systemic milieu) rather than that of few proteins.^64^ We recommend validating these proteins in an independent cohort of MCI and Alzheimer’s disease participants and a follow-up hypothesis-driven study focusing on specific molecular pathways and/or networks that are regulated by the serum analytes, which could provide better insights into how these proteins affect hippocampal cell fate and Alzheimer’s disease progression. One candidate is the p38 MAPK pathway, as its activation has been shown to trigger inhibition of proliferation, induce apoptosis and stimulate differentiation of progenitor cells.^65^

We recognize several limitations of our study. First, Alzheimer’s disease diagnosis was clinical only and none of the study participants had a post-mortem Alzheimer’s disease diagnosis. Second, there was no neuroimaging or CSF biomarker data available in the longitudinal cohort for us to ascertain the relationship between the altered neurogenic process and other Alzheimer’s disease-associated pathogenic processes. Third, our sample size and follow-up period were limited, despite the samples being drawn from two independent multicentre cohorts, and we lack data regarding potentially confounding lifestyle factors of the participants, such as physical activity levels. While the model has been cross-validated in our study, ideally, we and others will want to test our model in larger cohorts that include relevant lifestyle information. It will also be interesting to explore whether our results can be generalized to familial Alzheimer’s disease. Moreover, our cohort contained individuals with MCI with a high number of comorbidities, so having a larger cohort would enable us to study their influence on the neurogenic readouts, giving us more confidence that the readouts are reliable predictors of progression from MCI to Alzheimer’s disease. Fourth, we recognize that the assay we used in this study does not reconstitute the neurogenic niche in its entirety, and future experiments should see the expansion of this *in vitro* model to include other key players in Alzheimer’s disease, such as microglia or extend the duration of the assay to monitor synaptic formation and plasticity. Including other markers and characterizing at what point apoptosis increases in the differentiation phase may improve the prognostic accuracy of the assay. Fifth, we have previously shown that the cell line used in this assay contains single nucleotide polymorphisms that may reduce neurogenesis under inflammation,^66^ therefore, future studies should compare HPC lines with different genomic backgrounds or Alzheimer’s disease-specific induced pluripotent stem cell-derived neural progenitors. Sixth, the effects observed *in vitro* might not mirror those *in vivo* (i.e. foetal cells cultured with recombinant growth factors may not behave the same way as native adult neural precursors). Finally, although the strength of a serum assay is that serum is that blood collection is inexpensive and minimally invasive, it may not fully reflect the conditions of the brain milieu since the blood–brain barrier prevents free passage of molecules from the CNS to the blood.^67^ Therefore, a CSF assay may result in a higher prognostic accuracy.

In summary, the *in vitro* parabiosis assay presented in this study can model the effects of human systemic environment (i.e. serum) on HN. This assay can predict progression to Alzheimer’s disease up to 3.5 years before clinical diagnosis using a subset of baseline cellular readouts and years of education. Despite the limitations of this study, we believe that the proposed assay has the potential to facilitate early prognosis of Alzheimer’s disease and aid with effective stratification of study participants in clinical trials. The assay also presents a unique opportunity for us to facilitate our understanding of the potential mechanisms underlying alterations in human HN both in the context of health and disease.

## Supplementary Material

awac472_Supplementary_Data
