# Supplementary material for: Predicting progression to Alzheimer’s disease with human hippocampal progenitors exposed to serum
Source: Brain. 2023 Jan 27;146(5):2045–58. doi: 10.1093/brain/awac472 (PMC10151193; doi:10.1093/brain/awac472)
Supplement: awac472_Supplementary_Data [file awac472_supplementary_data.pdf]

# Supplementary material

# Predicting progression to Alzheimer’s disease with human hippocampal progenitors exposed to serum

Aleksandra Maruszak[1,†], Edina Silajdžić[1,†], Hyunah Lee[1,†], Tytus Murphy[1], Benjamine Liu[2], Liu Shi[2], Chiara de Lucia[1], Abdel Douiri[3], Evgenia Salta[4,5], Alejo J. Nevado[2], Charlotte E. Teunissen[5], Pieter J. Visser[6,7], Jack Price[1], Henrik Zetterberg[8-11], Simon Lovestone[2,12], and Sandrine Thuret[1]

[†]**These authors contributed equally to this work.**

## Abstract

Adult hippocampal neurogenesis (HN) is important for learning and memory and is altered early in Alzheimer’s disease. Since HN is modulated by the circulatory systemic environment, evaluating a proxy of how HN is affected by the systemic milieu could serve as an early biomarker for Alzheimer’s disease progression. Here, we used an *in vitro* assay to model the impact of systemic environment on HN. A human hippocampal progenitor cell line was treated with longitudinal serum samples from individuals with mild cognitive impairment (MCI), who either progressed to Alzheimer’s disease or remained cognitively stable. MCI to Alzheimer’s disease progression was characterised most prominently with decreased proliferation, increased cell death, and increased neurogenesis. A subset of ‘baseline’ cellular readouts together with education level were able to predict Alzheimer’s disease progression. The assay could provide a powerful platform for early prognosis, monitoring disease progression, and further mechanistic studies.

**Author affiliations:**

1 Department of Basic and Clinical Neuroscience, Institute of Psychiatry, Psychology & Neuroscience, King’s College London, London, UK

2 Department of Psychiatry, University of Oxford, UK

3 Department of Public Health Sciences, King’s College London, London, UK

4 Netherlands Institute for Neuroscience, Amsterdam, The Netherlands

5 Neurochemistry Lab and Biobank, Department of Clinical Chemistry, Amsterdam Neuroscience, VU University Medical Center, The Netherlands

6 Department of Psychiatry and Neuropsychology, Alzheimer Center Limburg, School for Mental Health and Neuroscience, Maastricht University, Maastricht, The Netherlands

7 Department of Neurology, Alzheimer Center, VU University Medical Center, Amsterdam, The Netherlands

8 Clinical Neurochemistry Laboratory, Sahlgrenska University Hospital, S-431 80, Mölndal, Sweden

9 Department of Neurodegenerative Disease, UCL Institute of Neurology, WC1N 3BG, London, UK

10 Institute of Neuroscience and Physiology, Department of Psychiatry and Neurochemistry, the Sahlgrenska Academy at the University of Gothenburg, S-431 80 Mölndal, Sweden

11 UK Dementia Research Institute at UCL, WC1E 6BT, London, UK

12 Janssen Medical UK, Turnhoutseweg 30, B-2340 Beerse, Belgium

Correspondence to: Sandrine Thuret

Maurice Wohl Clinical Neuroscience Institute, Department of Basic and Clinical Neuroscience, Institute of Psychiatry, Psychology & Neuroscience, King's College London, London, UK

sandrine.1.thuret@kcl.ac.uk

**Running title**: Alzheimer's disease prediction via serum

**Keywords:** Alzheimer's disease; prognostic biomarker; neurogenesis; hippocampal progenitors

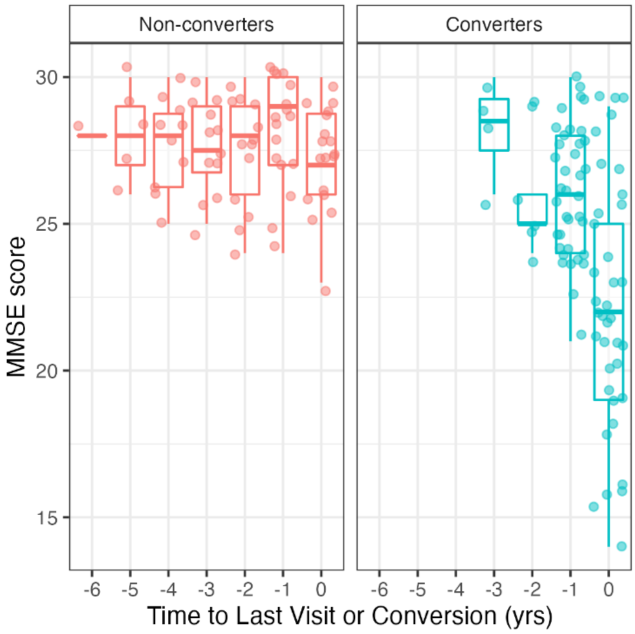


| Time (yrs) | -6 | -5 | -4 | -3 | -2 | -1 | 0 |
|---|---|---|---|---|---|---|---|
| n = 18 | 1 | 5 | 10 | 12 | 13 | 17 | 18 |
| n = 38 | 0 | 0 | 0 | 4 | 5 | 37 | 37 |

**Supplementary Figure 1. Mini Mental State Examination (MMSE) scores of participants over time.**

The box-and whisker plot, overlaid with a scatter of individual data points, shows changes in MMSE scores of participants over time, with the number of subjects in each group shown below the plot.

| Image Acquisition | |
|---|---|
| Objective | 10x |
| Camera Name | QUANTIX_32;1.00 |
| Acquisition Camera Mode | Standard (1024x1024;2x2) |
| AutoFocus Camera Mode | Standard (1024x1024;2x2) |
| AutoFocus Field Interval | 1 |

| AutoFocus Parameters | |
|---|---|
| Fine Focus Step Size | 17.6 |
| Fine Focus Plane Count | 9 |
| Coarse Focus Step Size | 70.4 |
| Coarse Focus Plane Count | 9 |
| Smart Focus Plane Count | 21 |
| Use Extended Range Focusing | False |
| Apply Backlash Correction | False |
| AutoFocus Method | STANDARD |
| Use Relaxed Pass/Fail Criteria | False |
| Focus Edge Threshhold | 0 |
| Focus Adjustment | 0 |
| Focus Score Min Ratio | 0.2 |
| Focus Score Mid Ratio | 0.4 |
| Focus Score Max Ratio | 0.5 |
| Focus Exposure Time for AutoExpose (seconds) | 0.1 |

| Scan Limits | |
|---|---|
| Max Fields for Well | 15 |
| Min Objects for Well | No Limit |
| Max Sparse Fields for Well | 3 |
| Min Objects for Field | 10 |
| Max Sparse Wells for Plate | 96 |

| Channel 1: Nuclei | | |
|---|---|---|
| Dye | BGRFR_386_23 | |
| Apply Illumination Correction | False | |
| Apply Background Correction | True | |
| Gain | 2 | |
| Z Offset | 0.00 | |
| Step Size | 11.67 | |
| Number of Steps | 3 | |
| Projection Method | | |
| Projection Direction | None | |
| Detection Mode | Widefield | |
| Grid Type | | |
| Pin Hole Size | | |
| Intensity Percent | 100 | |
| **Exposure Parameters** | | |
| Method | Fixed | |
| Exposure Time (seconds) | 0.015 | |
| **Object Identification** | | |
| Method | FixedThreshold | |
| Value | 50 | |
| **Object Selection Parameter** | **Min** | **Max** |
| ObjectShapeLWRCh1 | 0 | 1000 |
| ObjectAvgIntenCh1 | 0 | 65535 |
| ObjectVarIntenCh1 | 0 | 65535 |
| ObjectTotalIntenCh1 | 0 | 1000000000000 |
| ObjectAreaCh1 | 30 | 290 |
| ObjectShapeP2ACh1 | 0 | 1000 |

| Channel 3: ki67 | | |
|---|---|---|
| Dye | BGRFR_549_15 | |
| Apply Illumination Correction | False | |
| Apply Background Correction | True | |
| Gain | 2 | |
| Z Offset | 0.00 | |
| Step Size | 0.00 | |
| Number of Steps | 0 | |
| Projection Method | | |
| Projection Direction | None | |
| Detection Mode | Widefield | |
| Grid Type | | |
| Pin Hole Size | | |
| Intensity Percent | 100 | |
| **Exposure Parameters** | | |
| Method | Fixed | |
| Exposure Time (seconds) | 0.08 | |
| **Object Identification** | | |
| Method | None | |
| Value | 0 | |
| **Object Selection Parameter** | **Min** | **Max** |
| AvgIntenCh3 | 0 | 65535 |
| TotalIntenCh3 | 0 | 1000000000000 |
| AvgIntenCh3LevelHigh | 50 | |

| Channel 2: cc3 | | |
|---|---|---|
| Dye | BGRFR_485_20 | |
| Apply Illumination Correction | False | |
| Apply Background Correction | True | |
| Gain | 2 | |
| Z Offset | 0.00 | |
| Step Size | 0.00 | |
| Number of Steps | 0 | |
| Projection Method | | |
| Projection Direction | None | |
| Detection Mode | Widefield | |
| Grid Type | 0 | |
| Pin Hole Size | 0 | |
| Intensity Percent | 100 | |
| **Exposure Parameters** | | |
| Method | Fixed | |
| Exposure Time (seconds) | 0.15 | |
| **Object Identification** | | |
| Method | None | |
| Value | 0 | |
| **Object Selection Parameter** | **Min** | **Max** |
| AvgIntenCh2 | 0 | 65535 |
| TotalIntenCh2 | 0 | 1000000000000 |
| TargetAvgIntenCh2LevelHigh | 200 | |

| Channel 2: dcx | | |
|---|---|---|
| Dye | BGRFR_485_20 | |
| Apply Illumination Correction | False | |
| Apply Background Correction | True | |
| Gain | 2 | |
| Z Offset | 0.00 | |
| Step Size | 0.00 | |
| Number of Steps | 0 | |
| Projection Method | | |
| Projection Direction | None | |
| Detection Mode | Widefield | |
| Grid Type | 0 | |
| Pin Hole Size | 0 | |
| Intensity Percent | 100 | |
| **Exposure Parameters** | | |
| Method | Fixed | |
| Exposure Time (seconds) | 0.01 | |
| **Object Identification** | | |
| Method | None | |
| Value | 0 | |
| **Object Selection Parameter** | **Min** | **Max** |
| AvgIntenCh2 | 0 | 65535 |
| TotalIntenCh2 | 0 | 1000000000000 |
| TargetAvgIntenCh2LevelHigh | 150 | |

| Channel 3: map2 | | |
|---|---|---|
| Dye | BGRFR_549_15 | |
| Apply Illumination Correction | False | |
| Apply Background Correction | True | |
| Gain | 2 | |
| Z Offset | 0.00 | |
| Step Size | 0.00 | |
| Number of Steps | 0 | |
| Projection Method | | |
| Projection Direction | None | |
| Detection Mode | Widefield | |
| Grid Type | 0 | |
| Pin Hole Size | 0 | |
| Intensity Percent | 100 | |
| **Exposure Parameters** | | |
| Method | Fixed | |
| Exposure Time (seconds) | 0.06 | |
| **Object Identification** | | |
| Method | None | |
| Value | 0 | |
| **Object Selection Parameter** | **Min** | **Max** |
| AvgIntenCh3 | 0 | 65535 |
| TotalIntenCh3 | 0 | 1000000000000 |
| TargetAvgIntenCh3LevelHigh | 125 | |

**Supplementary Figure 2. Cellular phenotyping protocol on Thermo Scientific™ CellInsight™ CX5 High Content Screening Platform.**

Parameters used for semi-automated quantification of DAPI (nuclear), Ki67, CC3, DCX, and MAP2 are shown.

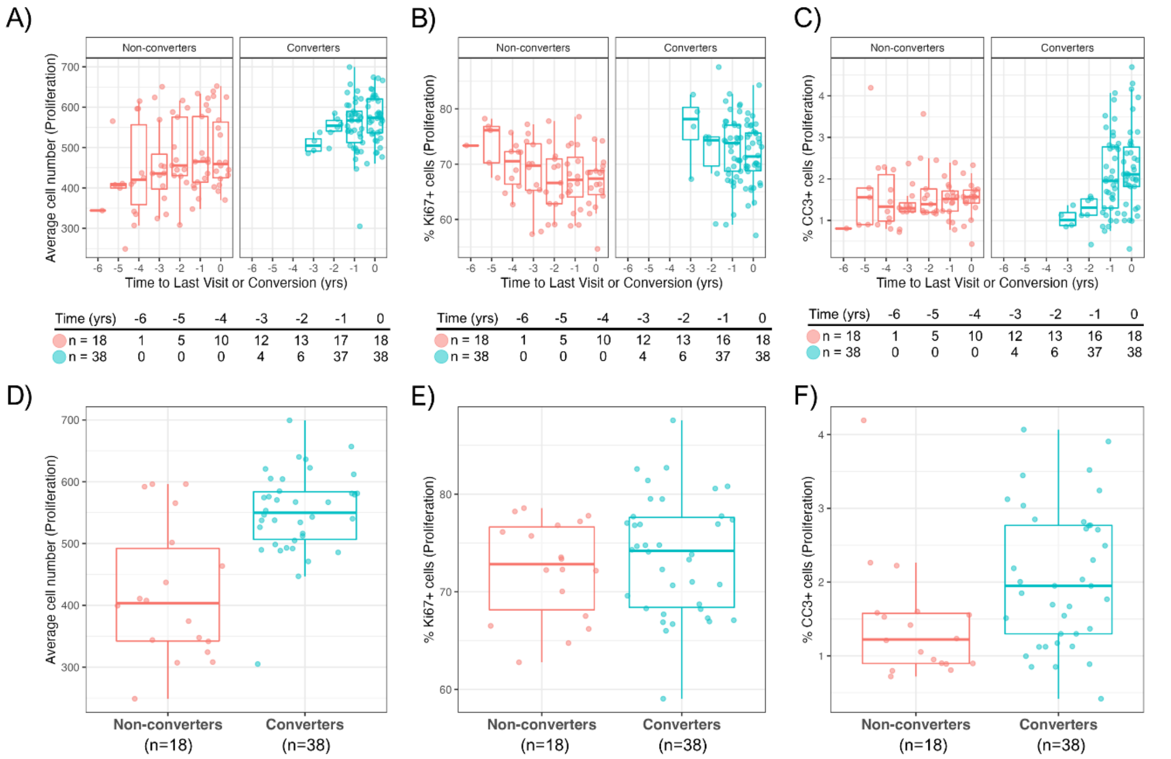


<u>**Supplementary Figure 3. Average cell number, proliferation, and apoptosis during the proliferation phase of the assay. (related to Fig. 2 and 3)**</u>

**A-C)** Box-and-whisker plots show the maximum, third quartile, median, first quartile, and minimum data for each measurement, overlaid with a scatter of individual data points showing the spread of the data over time, with the number of subjects in each group shown below the graph. Average cell count per field of view (total 45 fields of view, 15 fields per technical replicate) **(A)**, percentage of Ki67-positive cells **(B)**, percentage of CC3-positive cells **(C)**.

**D-F)** Box-and whisker plots overlaid with a scatter of individual data points showing the spread of the data at baseline, with the number of subjects in each group shown below the plot. Average cell count per field of view at baseline **(D)**, percentage of Ki67-positive cells at baseline **(E)**, percentage of CC3-positive cells at baseline **(F)**. Each dot represents mean of technical triplicates for each individual.

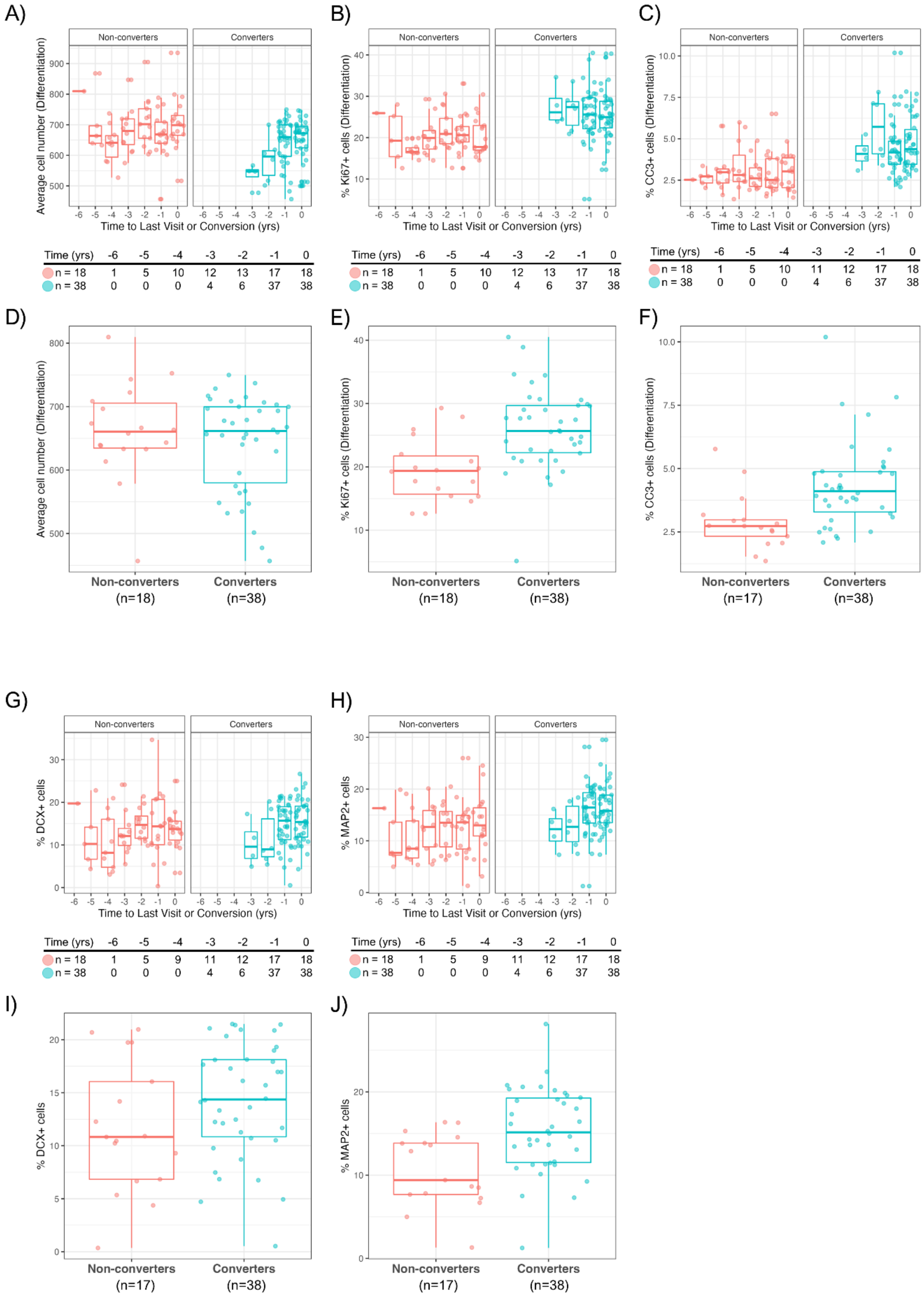


(figure legend on next page)

**Supplementary Figure 4. Average cell number, proliferation, and apoptosis during the differentiation phase of the assay. (related to Fig. 2 and 3)**

**A-C, G, H)** Box-and-whisker plots show the maximum, third quartile, median, first quartile, and minimum data for each measurement, overlaid with a scatter of individual data points showing the spread of the data over time, with the number of subjects in each group shown below the graph. Average cell count per field of view (total 45 fields of view, 15 fields per technical replicate) **(A)**, percentage of Ki67-positive cells **(B)**, percentage of CC3-positive cells **(C)**, percentage of DCX-positive cells **(G)**, percentage of MAP2-positive cells **(H)**.

**D-F, I, J)** Box-and whisker plots overlaid with a scatter of individual data points showing the spread of the data at baseline, with the number of subjects in each group shown below the plot. Average cell count per field of view at baseline **(D)**, percentage of Ki67-positive cells at baseline **(E)**, percentage of CC3-positive cells at baseline **(F)**, percentage of DCX-positive cells at baseline **(I)**, percentage of MAP2-positive cells at baseline **(J)**. Each dot represents mean of technical triplicates for each individual.

```
library(lme4)
library(dplyr)

### CC3 ###
data <- full_data %>%
  group_by(group, ID) %>%
  dplyr::filter(group==1) %>%
  dplyr::select(ID, group,
                CC3_prol,
                time_to_last_visit_or_conversion,
                edu10.5, supplement_intake) %>% drop_na()
fml <- formula(CC3_prol ~ (1|ID))
mod0 <- lmer(fml, data)
fml <- formula(CC3_prol ~ time_to_last_visit_or_conversion + (1|ID))
mod1 <- lmer(fml, data)
fml <- formula(CC3_prol ~ time_to_last_visit_or_conversion + edu10.5 +
(1|ID))
mod2 <- lmer(fml, data)
fml <- formula(CC3_prol ~ time_to_last_visit_or_conversion + edu10.5 +
supplement_intake + (1|ID))
mod3 <- lmer(fml, data)
fml <- formula(CC3_prol ~ time_to_last_visit_or_conversion + edu10.5 +
supplement_intake + edu10.5*supplement_intake + (1|ID))
mod4 <- lmer(fml, data)
anova(mod0, mod1, mod2, mod3, mod4)
```

```
## refitting model(s) with ML (instead of REML)
```

```
## Data: data
## Models:
## mod0: CC3_prol ~ (1 | ID)
## mod1: CC3_prol ~ time_to_last_visit_or_conversion + (1 | ID)
## mod2: CC3_prol ~ time_to_last_visit_or_conversion + edu10.5 + (1 | ID)
## mod3: CC3_prol ~ time_to_last_visit_or_conversion + edu10.5 +
supplement_intake + (1 | ID)
## mod4: CC3_prol ~ time_to_last_visit_or_conversion + edu10.5 +
supplement_intake + edu10.5 * supplement_intake + (1 | ID)
##      npar    AIC    BIC  logLik deviance  Chisq Df Pr(>Chisq)
## mod0    3 177.05 184.38 -85.527   171.05
## mod1    4 174.18 183.95 -83.088   166.18 4.8782  1    0.02720 *
## mod2    5 172.05 184.26 -81.025   162.05 4.1251  1    0.04225 *
## mod3    6 173.64 188.30 -80.821   161.64 0.4093  1    0.52234
## mod4    7 175.49 192.59 -80.746   161.49 0.1489  1    0.69961
## ---
## Signif. codes:  0 '***' 0.001 '**' 0.01 '*' 0.05 '.' 0.1 ' ' 1
```

```
### DCX ###
data <- full_data %>%
  group_by(group, ID) %>%
  dplyr::filter(group==1) %>%
  dplyr::select(ID, group,
                DCX_diff,
                time_to_last_visit_or_conversion,
                MMSE_baseline, AD_drugs) %>% drop_na()
fml <- formula(DCX_diff ~ (1|ID))
mod0 <- lmer(fml, data)
fml <- formula(DCX_diff ~ time_to_last_visit_or_conversion + (1|ID))
mod1 <- lmer(fml, data)
fml <- formula(DCX_diff ~ time_to_last_visit_or_conversion + MMSE_baseline
+ (1|ID))
mod2 <- lmer(fml, data)
fml <- formula(DCX_diff ~ time_to_last_visit_or_conversion + MMSE_baseline
+ AD_drugs + (1|ID))
mod3 <- lmer(fml, data)
```

```
fml <- formula(DCX_diff ~ time_to_last_visit_or_conversion + MMSE_baseline
+ AD_drugs + MMSE_baseline*AD_drugs + (1|ID))
mod4 <- lmer(fml, data)
anova(mod0, mod1, mod2, mod3, mod4)
```

```
## refitting model(s) with ML (instead of REML)
```

```
## Data: data
## Models:
## mod0: DCX_diff ~ (1 | ID)
## mod1: DCX_diff ~ time_to_last_visit_or_conversion + (1 | ID)
## mod2: DCX_diff ~ time_to_last_visit_or_conversion + MMSE_baseline + (1 |
ID)
## mod3: DCX_diff ~ time_to_last_visit_or_conversion + MMSE_baseline +
AD_drugs + (1 | ID)
## mod4: DCX_diff ~ time_to_last_visit_or_conversion + MMSE_baseline +
AD_drugs + MMSE_baseline * AD_drugs + (1 | ID)
##      npar    AIC    BIC  logLik deviance  Chisq Df Pr(>Chisq)
## mod0    3 497.84 505.13 -245.92   491.84
## mod1    4 493.22 502.94 -242.61   485.22 6.6177  1   0.010097 *
## mod2    5 488.00 500.15 -239.00   478.00 7.2214  1   0.007204 **
## mod3    6 489.64 504.23 -238.82   477.64 0.3586  1   0.549261
## mod4    7 489.97 506.98 -237.98   475.97 1.6749  1   0.195608
## ---
## Signif. codes:  0 '***' 0.001 '**' 0.01 '*' 0.05 '.' 0.1 ' ' 1
```

```
### MAP2 ###
data <- full_data %>%
  group_by(group, ID) %>%
  dplyr::filter(group==1) %>%
  dplyr::select(ID, group,
                MAP2_diff,
                time_to_last_visit_or_conversion,
                female, APOE4) %>% drop_na()
fml <- formula(MAP2_diff ~ (1|ID))
mod0 <- lmer(fml, data)
fml <- formula(MAP2_diff ~ time_to_last_visit_or_conversion + (1|ID))
mod1 <- lmer(fml, data)
fml <- formula(MAP2_diff ~ time_to_last_visit_or_conversion + female +
(1|ID))
mod2 <- lmer(fml, data)
fml <- formula(MAP2_diff ~ time_to_last_visit_or_conversion + female +
APOE4 + (1|ID))
mod3 <- lmer(fml, data)
fml <- formula(MAP2_diff ~ time_to_last_visit_or_conversion + female +
APOE4 + female*APOE4 + (1|ID))
mod4 <- lmer(fml, data)
anova(mod0, mod1, mod2, mod3, mod4)
```

```
## refitting model(s) with ML (instead of REML)
```

```
## Data: data
## Models:
## mod0: MAP2_diff ~ (1 | ID)
## mod1: MAP2_diff ~ time_to_last_visit_or_conversion + (1 | ID)
## mod2: MAP2_diff ~ time_to_last_visit_or_conversion + female + (1 | ID)
## mod3: MAP2_diff ~ time_to_last_visit_or_conversion + female + APOE4 + (1
| ID)
## mod4: MAP2_diff ~ time_to_last_visit_or_conversion + female + APOE4 +
female * APOE4 + (1 | ID)
##      npar    AIC    BIC  logLik deviance  Chisq Df Pr(>Chisq)
## mod0    3 450.73 457.84 -222.37   444.73
## mod1    4 449.61 459.09 -220.81   441.61 3.1185  1    0.07741 .
## mod2    5 445.75 457.60 -217.87   435.75 5.8641  1    0.01545 *
## mod3    6 446.81 461.03 -217.40   434.81 0.9398  1    0.33232
```

```
## mod4     7 448.33 464.92 -217.17   434.33 0.4752  1    0.49060
## ---
## Signif. codes:  0 '***' 0.001 '**' 0.01 '*' 0.05 '.' 0.1 ' ' 1
```

**Supplementary Figure 5. No significant interaction between explanatory variables in linear mixed-effects models of converters only dataset. (related to Fig. 2A-D)**

The results of linear mixed-effects modelling generated with the R package knitr (version 1.40) show no significant interaction between explanatory variables (Pr(>Chisq) > 0.05 when compared with the null model). Biologically plausible interactions were tested between education level (dichotomised at 10.5 yrs) and supplement intake when apoptosis (% CC3+) during proliferation was the response variable; between baseline MMSE scores and AD drug intake when the number of neuroblasts (% DCX+) was the response variable; and between sex (female assigned 1) and APOE4 status when the number of mature neurons (% MAP2+) was the response variable. Bold texts (i.e., mod3 and mod4) indicate the models in which the interaction of explanatory variables was tested. The report was generated with the R package knitr (version 1.40) using the command *knitr::stitch_rhtml('filename.r')*.

A)

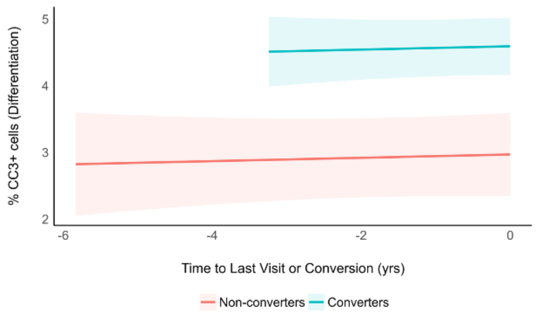


C)

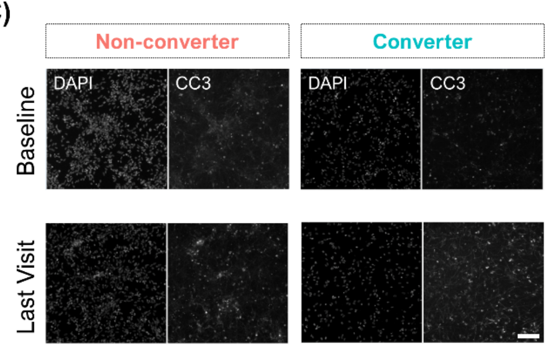


B)

| % CC3+ cells (Differentiation) | Null | | | | Random intercept (Pr>Chisq) < 0.001 | | | |
|---|---|---|---|---|---|---|---|---|
| *Predictors* | *Estimates* | *std. Error* | *CI* | *p* | *Estimates* | *std. Error* | *CI* | *p* |
| **(Intercept)** | 4.02 | 0.2 | 3.62 – 4.42 | <0.001 | **2.97** | **0.32** | **2.35 – 3.59** | **<0.001** |
| **MCI to AD progression** | | | | | **1.62** | **0.37** | **0.89 – 2.36** | **<0.001** |
| Time to Last Visit or Conversion (yrs) | | | | | 0.02 | 0.06 | -0.09 – 0.14 | 0.68 |
| Marginal R2 / Conditional R2 | 0.000 / 0.733 | | | | 0.243 / 0.737 | | | |
| Deviance | 515.084 | | | | 497.247 | | | |
| AIC | 522.443 | | | | 512.882 | | | |
| log-Likelihood | -258.221 | | | | -251.441 | | | |

**Supplementary Figure 6. Apoptosis during differentiation assay between non-converters and converters.**

**A, B)** Modelled trajectories **(A)** and results of the linear mixed-effects regression model **(B)** fitted to the apoptosis during differentiation assay dataset. Either time of last visit (for non-converters; turquoise) or time of conversion to AD (for converters; red) was assigned 0, and the number of years before that were assigned negative values (i.e., one year before conversion is -1). The effect of MCI to AD progression was significant ($p < 0.001$) and positive (beta = 1.62), while time to last visit or conversion (yrs) did not have any significant effect as an explanatory variable on %CC3+ cells during differentiation ($p = 0.68$).

**C)** Representative images of differentiation phase cells treated with serum from the same individual. Left (non-converter panel, ID: LND008): serum sample taken at baseline (5.83 years before last visit) and last visit. Right (converter panel, ID: LND018): serum sample taken at baseline (3 years before last visit) and the time of conversion to AD (equals last visit). Nuclei are stained with DAPI. Cleaved caspase 3 (CC3) was used to label apoptotic cells. Scale bar 100 μm.

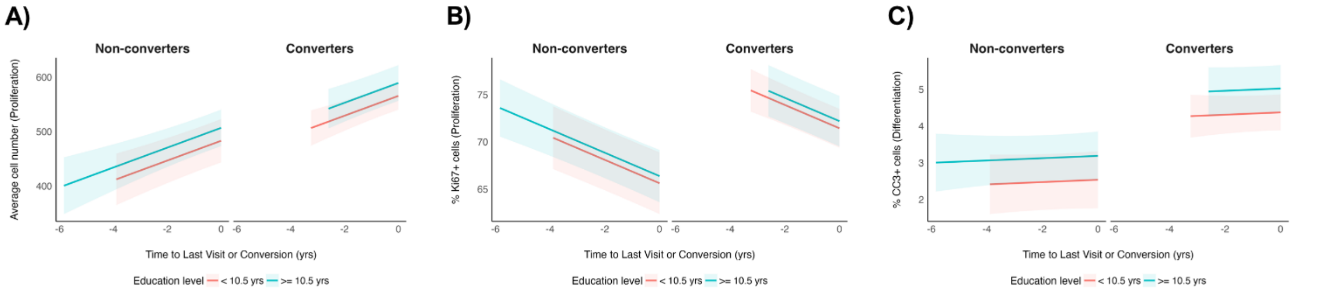


**A)**

| | *Estimates* | *std. Error* | *CI* | *p* |
|---|---|---|---|---|
| (Intercept) | 483.30 | 20.09 | 443.61 – 523.00 | **<0.001** |
| MCI to AD progression | 82.47 | 19.96 | 43.03 – 121.90 | **<0.001** |
| Time to Last Visit or Conversion (yrs) | 18.22 | 3.75 | 10.81 – 25.63 | **<0.001** |
| Education level [>= 10.5 yrs] | 23.75 | 18.79 | -13.37 – 60.87 | 0.208 |

**B)**

| | *Estimates* | *std. Error* | *CI* | *p* |
|---|---|---|---|---|
| (Intercept) | 65.62 | 1.65 | 62.36 – 68.88 | **<0.001** |
| MCI to AD progression | 5.82 | 1.63 | 2.60 – 9.05 | **<0.001** |
| Time to Last Visit or Conversion (yrs) | -1.24 | 0.18 | -1.59 – -0.89 | **<0.001** |
| Education level [>= 10.5 yrs] | 0.76 | 1.53 | -2.27 – 3.79 | 0.623 |

**C)**

| | *Estimates* | *std. Error* | *CI* | *p* |
|---|---|---|---|---|
| (Intercept) | 2.54 | 0.39 | 1.77 – 3.31 | **<0.001** |
| MCI to AD progression | 1.83 | 0.38 | 1.08 – 2.59 | **<0.001** |
| Time to Last Visit or Conversion (yrs) | 0.03 | 0.06 | -0.09 – 0.15 | 0.603 |
| Education level [>= 10.5 yrs] | 0.65 | 0.36 | -0.06 – 1.36 | 0.074 |

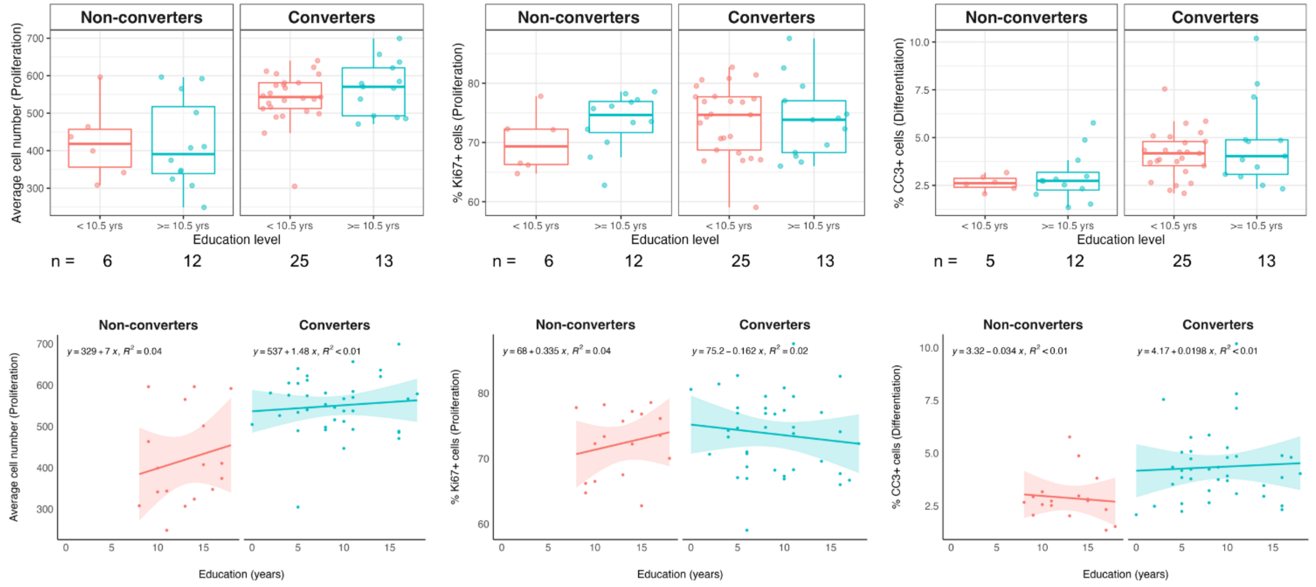


(figure legend on next page)

**Supplementary Figure 7. No effect of education on neurogenesis assay predictors of MCI to AD progression.**

**Top:** results of linear mixed-effects models fitted to the longitudinal dataset, when the response variables were either average cell number during proliferation **(A)**, % Ki67+ cells during proliferation **(B)**, or % CC3+ cells during differentiation **(C)**. The explanatory variables were 'time to last visit or conversion (yrs)', 'MCI to AD progression (converters assigned 1)', and 'education level (dichotomised at 10.5 yrs)'. None of the models indicate significant effects of education level ($p > 0.05$) on the response variables. Red: education < 10.5 years. Turquoise: education >= 10.5 years.

**Middle:** the box-and whisker plots, overlaid with a scatter of individual data points, showing the distribution of baseline average cell number during proliferation **(A)**, % Ki67+ cells during proliferation **(B)**, or % CC3+ cells during differentiation **(C)**, stratified according to 'MCI to AD progression (non-converters vs converters)' and 'education level (dichotomised at 10.5 yrs)'. The number of subjects in each sub-group is shown below the plots. Baseline levels of average cell number during proliferation **(A)**, % Ki67+ cells during proliferation **(B)**, and % CC3+ cells during differentiation **(C)** are similar between 'education < 10.5 yrs' and 'education >= 10.5 yrs' groups, regardless of 'MCI to AD progression' status. Red: education < 10.5 years. Turquoise: education >= 10.5 years.

**Bottom:** the distribution of baseline average cell number during proliferation **(A)**, % Ki67+ cells during proliferation **(B)**, or % CC3+ cells during differentiation **(C)** plotted against education in years (not dichotomised), stratified according to 'MCI to AD progression (non-converters vs converters)'. Linear regression lines were fitted to the data. The equations and R-squared values are shown in each plot. All R-squared values are less than 0.1 indicating that education in years have little or no explanatory value.

**Supplementary Table 1. Cell culture medium components.**

| Component | Concentration | Supplier (Catalogue number) |
|---|---|---|
| Dulbecco’s Modified Eagle’s Medium/Nutrient Mixture F-12 Ham | | Sigma Aldrich (D6421) |
| Albumin, human | 0.03% | Zenlab 20 |
| apo-Transferrin, human | 100 µg/ml | Sigma Aldrich (T1147) |
| Epidermal growth factor | 20 ng/ml | PeproTech (AF-100-15) |
| Fibroblast growth factor-basic | 10 ng/ml | PeproTech (100-18B) |
| Insulin, human recombinant | 5 µg/ml | Sigma Aldrich (I9278) |
| L-glutamine | 2 mM | Sigma Aldrich (G7513) |
| Progesterone | 60 ng/ml | Sigma Aldrich (P8783) |
| Putrescine dihydrochloride | 16.2 µg/ml | Sigma Aldrich (P5780) |
| Sodium selenite | 40 ng/ml | Sigma Aldrich (S9133) |

**Supplementary Table 2. Source data table. (related to Fig. 2-5)**

Available on figshare (https://doi.org/10.6084/m9.figshare.19778941)

**Supplementary Table 3. Identifiers of all proteins quantified in SomaScan. (related to Fig. 5)**

Available on figshare (https://doi.org/10.6084/m9.figshare.19778938)

**Supplementary Table 4. Significant predictors of proliferation phase readouts from MCI converters only. (related to Fig. 2A-D)** Models with the lowest Akaike Information Criterion (AIC) and deviance were selected as the best fit. Coefficient estimates, standard errors, 95% confidence intervals (CI) around the regression coefficient, and significance levels for all predictors in the analysis are provided.

| **Average cell number** | Null | | | | Random intercept (model 1) Pr(>Chisq) = 0.002 | | | |
|---|---|---|---|---|---|---|---|---|
| Predictors | Estimates | std. Error | 95% CI | p | Estimates | std. Error | 95% CI | p |
| (Intercept) | 561.95 | 9.14 | 543.77 – 580.12 | <0.001 | 573.52 | 9.75 | 554.12 – 592.93 | <0.001 |
| **Time to Conversion** | | | | | **17.37** | **5.28** | **6.87 – 27.87** | **0.001** |
| Deviance | 919.997 | | | | 909.978 | | | |
| AIC | 919.741 | | | | 906.582 | | | |
| log-Likelihood | -456.87 | | | | -449.291 | | | |

| **% Ki67+ cells** | Null | | | | Random intercept (model 1) Pr(>Chisq) < 0.001 | | | |
|---|---|---|---|---|---|---|---|---|
| Predictors | Estimates | std. Error | 95% CI | p | Estimates | std. Error | 95% CI | p |
| (Intercept) | 72.52 | 0.92 | 70.69 – 74.35 | <0.001 | 71.58 | 0.93 | 69.72 – 73.44 | <0.001 |
| **Time to Conversion** | | | | | **-1.44** | **0.29** | **-2.03 – -0.86** | **<0.001** |
| Deviance | 482.021 | | | | 461.909 | | | |
| AIC | 486.357 | | | | 468.894 | | | |
| log-Likelihood | -240.178 | | | | -230.447 | | | |

| **% CC3+ cells** | Null | | | | Random intercept (model 1) Pr(>Chisq) = 0.027 | | | | Random intercept (model 2) Pr(>Chisq) = 0.042 | | | |
|---|---|---|---|---|---|---|---|---|---|---|---|---|
| Predictors | Estimates | std. Error | 95% CI | p | Estimates | std. Error | 95% CI | p | Estimates | std. Error | 95% CI | p |
| (Intercept) | 2.16 | 0.15 | 1.86 – 2.46 | <0.001 | 2.24 | 0.15 | 1.94 – 2.54 | <0.001 | 2.45 | 0.18 | 2.09 – 2.80 | <0.001 |
| **Time to Conversion** | | | | | **0.12** | **0.05** | **0.01 – 0.23** | **0.029** | **0.12** | **0.05** | **0.01 – 0.23** | **0.031** |
| **Education (>= 10.5 yrs)** | | | | | | | | | **-0.6** | **0.3** | **-1.20 – -0.01** | **0.046** |
| Deviance | 171.061 | | | | 166.176 | | | | 162.063 | | | |
| AIC | 179.018 | | | | 180.158 | | | | 178.743 | | | |
| log-Likelihood | -86.509 | | | | -86.079 | | | | -84.372 | | | |

**Supplementary Table 5. Significant predictors of differentiation phase readouts from MCI converters only. (related to Fig. 2E-H)** Models with the lowest Akaike Information Criterion (AIC) and deviance were selected as the best fit. Coefficient estimates, standard errors, 95% confidence intervals (CI) around the regression coefficient, and significance levels for all predictors in the analysis are provided.

| **Average cell number** | Null | | | | Random intercept (model 1) Pr(>Chisq) = 0.015 | | | |
|---|---|---|---|---|---|---|---|---|
| Predictors | Estimates | std. Error | 95% CI | p | Estimates | std. Error | 95% CI | p |
| (Intercept) | 647.1 | 10.73 | 625.75 – 668.44 | <0.001 | 655.13 | 10.91 | 633.41 – 676.84 | <0.001 |
| **Time to Conversion** | | | | | **12.17** | **4.99** | **2.24 – 22.11** | **0.017** |
| Deviance | 920.072 | | | | 914.187 | | | |
| AIC | 919.493 | | | | 910.635 | | | |
| log-Likelihood | -456.747 | | | | -451.318 | | | |

| **% DCX+ cells** | Null | | | | Random intercept (model 1) Pr(>Chisq) = 0.010 | | | | Random intercept (model 2) Pr(>Chisq) = 0.007 | | | |
|---|---|---|---|---|---|---|---|---|---|---|---|---|
| Predictors | Estimates | std. Error | 95% CI | p | Estimates | std. Error | 95% CI | p | Estimates | std. Error | 95% CI | p |
| (Intercept) | 15.01 | 0.77 | 13.47 – 16.54 | <0.001 | 15.78 | 0.82 | 14.15 – 17.42 | <0.001 | 42.33 | 9.71 | 23.00 – 61.66 | <0.001 |
| **Time to Conversion** | | | | | **1.22** | **0.47** | **0.29 – 2.15** | **0.01** | **1.20** | **0.46** | **0.27 – 2.12** | **0.012** |
| **MMSE baseline (>= 27)** | | | | | | | | | **-3.91** | **1.43** | **-6.75 – -1.08** | **0.007** |
| Deviance | 491.847 | | | | 485.222 | | | | 478.007 | | | |
| AIC | 496.536 | | | | 491.636 | | | | 484.080 | | | |
| log-Likelihood | -245.268 | | | | -241.818 | | | | -237.040 | | | |

| **% MAP2+ cells** | Null | | | | Random intercept (model 1) Pr(>Chisq) = 0.032 | | | | Random intercept (model 2) Pr(>Chisq) = 0.008 | | | |
|---|---|---|---|---|---|---|---|---|---|---|---|---|
| Predictors | Estimates | std. Error | 95% CI | p | Estimates | std. Error | 95% CI | p | Estimates | std. Error | 95% CI | p |
| (Intercept) | 16.05 | 0.68 | 14.69 – 17.40 | <0.001 | 16.66 | 0.73 | 15.21 – 18.10 | <0.001 | 18.72 | 1.02 | 16.69 – 20.76 | <0.001 |
| **Time to Conversion** | | | | | **0.92** | **0.43** | **0.07 – 1.76** | **0.035** | **0.95** | **0.43** | **0.10 – 1.79** | **0.029** |
| **Female** | | | | | | | | | **-3.39** | **1.26** | **-5.90 – -0.89** | **0.009** |
| Deviance | 480.081 | | | | 475.482 | | | | 468.521 | | | |
| AIC | 485.019 | | | | 482.343 | | | | 475.283 | | | |
| log-Likelihood | -239.51 | | | | -237.171 | | | | -232.641 | | | |

**Supplementary Table 6. Significant predictors of proliferation phase readouts from both MCI converters and non-converters. (related to Fig. 3A-B)** Models with the lowest Akaike Information Criterion (AIC) and deviance were selected as the best fit. Coefficient estimates, standard errors, 95% confidence intervals (CI) around the regression coefficient, and significance levels for all predictors in the analysis are provided.

| **Average cell number** | Null | | | | Random intercept (model 1) Pr(>Chisq) < 0.001 | | | | Random slope (model 2) Pr(>Chisq) = 0.033 | | | |
|---|---|---|---|---|---|---|---|---|---|---|---|---|
| Predictors | Estimates | std. Error | 95% CI | p | Estimates | std. Error | 95% CI | p | Estimates | std. Error | 95% CI | p |
| (Intercept) | 530.78 | 10.93 | 509.20 – 552.37 | <0.001 | 498.54 | 16.67 | 465.60 – 531.48 | <0.001 | 499.18 | 15.77 | 468.02 – 530.34 | <0.001 |
| **MCI to AD progression** | | | | | **74.79** | **19.83** | **35.62 – 113.95** | **<0.001** | **74.71** | **19.11** | **36.96 – 112.46** | **<0.001** |
| **Time to conversion or last visit** | | | | | **17.08** | **2.71** | **11.71 – 22.44** | **<0.001** | **18.23** | **3.76** | **10.81 – 25.65** | **<0.001** |
| Deviance | 1806.828 | | | | 1753.376 | | | | 1746.612 | | | |
| AIC | 1806.21 | | | | 1745.491 | | | | 1742.197 | | | |
| log-Likelihood | -900.105 | | | | -867.746 | | | | -864.098 | | | |

| **% Ki67+ cells** | Null | | | | Random intercept (model 1) Pr(>Chisq) < 0.001 | | | |
|---|---|---|---|---|---|---|---|---|
| Predictors | Estimates | std. Error | 95% CI | p | Estimates | std. Error | 95% CI | p |
| (Intercept) | 71.11 | 0.76 | 69.61 – 72.61 | <0.001 | 66.12 | 1.29 | 63.57 – 68.67 | <0.001 |
| **MCI to AD progression** | | | | | **5.58** | **1.54** | **2.53 – 8.63** | **<0.001** |
| **Time to conversion or last visit** | | | | | **-1.24** | **0.18** | **-1.59 – -0.89** | **<0.001** |
| Deviance | 935.078 | | | | 887.001 | | | |
| AIC | 939.789 | | | | 894.786 | | | |
| log-Likelihood | -466.895 | | | | -442.393 | | | |

**Supplementary Table 7. Significant predictors of differentiation phase readouts from both MCI converters and non-converters. (related to Fig. 3C-D)** Models with the lowest Akaike Information Criterion (AIC) and deviance were selected as the best fit. Coefficient estimates, standard errors, 95% confidence intervals (CI) around the regression coefficient, and significance levels for all predictors in the analysis are provided.

| **Average cell number** | Null | | | | Random intercept (model 1) Pr(>Chisq) < 0.001 | | | |
|---|---|---|---|---|---|---|---|---|
| Predictors | Estimates | std. Error | 95% CI | p | Estimates | std. Error | 95% CI | p |
| (Intercept) | 658.6 | 9.13 | 640.57 – 676.63 | <0.001 | 701.94 | 16.06 | 670.22 – 733.66 | <0.001 |
| **MCI to AD progression** | | | | | **-47.64** | **18.98** | **-85.13 – -10.15** | **0.013** |
| **Time to conversion or last visit** | | | | | **11.48** | **2.95** | **5.65 – 17.32** | **<0.001** |
| Deviance | 1784.992 | | | | 1767.15 | | | |
| AIC | 1784.734 | | | | 1759.278 | | | |
| log-Likelihood | -889.367 | | | | -874.639 | | | |

| **% MAP2+ cells** | Null | | | | Random intercept (model 1) Pr(>Chisq) < 0.001 | | | |
|---|---|---|---|---|---|---|---|---|
| Predictors | Estimates | std. Error | 95% CI | p | Estimates | std. Error | 95% CI | p |
| (Intercept) | 14.82 | 0.6 | 13.64 – 16.00 | <0.001 | 13.45 | 1.02 | 11.44 – 15.46 | <0.001 |
| **MCI to AD progression** | | | | | **2.96** | **1.19** | **0.62 – 5.31** | **0.014** |
| **Time to conversion or last visit** | | | | | **0.56** | **0.23** | **0.11 – 1.02** | **0.015** |
| Deviance | 915.54 | | | | 900.735 | | | |
| AIC | 920.732 | | | | 909.081 | | | |
| log-Likelihood | -457.366 | | | | -449.54 | | | |

**Supplementary Table 8. 205 proteins significantly differentially expressed between MCI converters and non-converters. (related to Fig. 5A)**

Available on figshare (https://doi.org/10.6084/m9.figshare.19778911)

**Supplementary Table 9. A panel of 15 proteins that discriminate MCI converters from MCI non-converters. (related to Fig. 5C)** Using the least absolute shrinkage and selection operator (LASSO) and support vector machines with 10-times cross-validation, a panel of 15 proteins were found to be capable of discriminating MCI converters from MCI non-converters. q-value is the false discovery rate (FDR) corrected p-value based on Wilcoxon rank-sum test.

| UniProt ID | Beta coefficient | p-value | q-value | Protein name | Gene name |
|---|---|---|---|---|---|
| Q9NPH3 | 0.10683 | 0.00031 | 0.66869 | Interleukin-1 receptor accessory protein | *IL1RAP* |
| Q8TBE7 | 0.10915 | 0.00116 | 0.66869 | Solute carrier family 35 member G2 | *SLC35G2* |
| Q9UHD0 | 0.09371 | 0.00133 | 0.66869 | Interleukin-19 | *IL19* |
| Q9NTK1 | 0.08752 | 0.00152 | 0.66869 | Protein DEPP1 | *DEPP1* |
| Q8N474 | 0.16495 | 0.00197 | 0.66869 | Secreted frizzled-related protein 1 | *SFRP1* |
| P43251 | 0.10596 | 0.00327 | 0.66869 | Biotinidase | *BTD* |
| Q8NBP7 | 0.09953 | 0.00369 | 0.66869 | Proprotein convertase subtilisin/kexin type 9 | *PCSK9* |
| Q9UK55 | 0.08628 | 0.00785 | 0.66869 | Protein Z-dependent protease inhibitor | *SERPINA10* |
| Q6UWD8 | -0.08015 | 0.02362 | 0.90164 | Transmembrane protein C16orf54 | *C16orf54* |
| P19876.P19875 | -0.07721 | 0.02799 | 0.95018 | C-X-C motif chemokine 3 & C-X-C motif chemokine 2 | *CXCL3 & CXCL2* |
| Q9Y5Q6 | 0.04643 | 0.03844 | 0.95830 | Insulin-like peptide INSL5 | *INSL5* |
| Q96PU8 | -0.06558 | 0.03844 | 0.95830 | Protein quaking | *QKI* |
| P52907 | -0.10166 | 0.04017 | 0.95830 | F-actin-capping protein subunit alpha-1 | *CAPZA1* |
| P00797 | -0.07242 | 0.05646 | 0.95830 | Renin | *REN* |
| O14548 | 0.10303 | 0.09762 | 0.97017 | Cytochrome c oxidase subunit 7A-related protein, mitochondrial | *COX7A2L* |

**Supplementary Table 10. Pathway analysis (canonical pathways) (IPA) on 205 differentially expressed proteins in MCI converters.** The z-score reflects an overall predicted activation and inhibition state of the biological function. Positive and negative z-scores predict activation and inhibition, respectively.

| Pathway | molecules | z-score | p-value | ratio |
|---|---|---|---|---|
| Coagulation system | 7 | 0.378 | 1.92 E-04 | 7/26 |
| Acute phase response signalling | 12 | 2.121 (biased) | 3.45 E-03 | 12/100 |
| Extrinsic prothrombin activation pathway | 3 | | 1.11 E-02 | 3/10 |
| FXR/RXR activation | 7 | | 1.46 E-02 | 7/53 |
| Notch signalling | 3 | | 2.37 E-02 | 3/13 |
| Superpathway of methionine degradation | 2 | | 3.21 E-02 | 2/6 |
| Wnt/b-catenin signalling | 6 | - 0.447 | 3.53 E-02 | 6/50 |
| Role of osteoblasts, osteoclasts, and chondrocytes in rheumatoid arthritis | 9 | | 3.68 E-02 | 9/92 |
| EGF signalling | | 1 (biased) | 3.71 E-02 | 4/26 |
| Ceramide signalling | 4 | 1 (biased) | 4.71 E-02 | 4/28 |
| ATM signalling | 4 | 0 | 4.71 E-02 | 4/28 |
| IL-17A signalling in airway cells | 4 | 2 (biased) | 4.71 E-02 | 4/28 |

**Supplementary Table 11. Network Analysis (IPA) on 205 differentially expressed proteins in MCI converters. (related to Fig. 5D-F)** Network Scores are based on the hypergeometric distribution and are calculated with the right-tailed Fisher's Exact Test. Score ranks the networks based on the following information: 1) the degree of relevance of the network to the Focus molecules in the dataset, 2) the number of Focus molecules in the network, 3) the network size, 4) the total number of Focus molecules analysed, and 5) the total number of molecules in the QIAGEN Knowledge Base that could potentially be included in networks.

| Associated Network Functions | Score |
|---|---|
| Haematological System Development and Function, Organismal Functions, Organismal Injury and Abnormalities | 48 |
| Cell Death and Survival, Embryonic Development, Organismal Development | 43 |
| Cell-to-Cell Signalling and Interaction, Cellular Function and Maintenance, Inflammatory Response | 21 |
| DNA Replication, Recombination, and Repair, Cancer, Organismal Injury and Abnormalities | 17 |
| Organismal Survival, Connective Tissue Disorders, Developmental Disorder | 17 |
